# Targeting NSUN2‐Mediated m^5^C Modification Attenuates Chondrocyte Senescence and NLRP3 Activation in Osteoarthritis

**DOI:** 10.1002/advs.76370

**Published:** 2026-07-06

**Authors:** Guping Mao, Wei Li, Zhencan Lin, Zengfa Deng, Ming Li, Zongrui Jiang, Changzhao Li, Dianbo Long, Yan Kang

**Affiliations:** ^1^ Department of Sports Medicine the First Affiliated Hospital Sun Yat‐sen University Guangzhou Guangdong China; ^2^ Guangdong Provincial Key Laboratory of Orthopedics and Traumatology the First Affiliated Hospital Sun Yat‐sen University Guangzhou Guangdong China; ^3^ Department of Sports Medicine Shenzhen Second People's Hospital the First Affiliated Hospital of Shenzhen University Shenzhen Guangdong China; ^4^ Department of Orthopaedics General Hospital of Southern Theater Command Guangzhou Guangdong China

**Keywords:** IP3R3, m^5^C, NLRP3 inflammasome, NSUN2, Osteoarthritis, Senescence

## Abstract

Osteoarthritis (OA) is a prevalent age‐related disease associated with significant pain and disability. Although 5‐methylcytosine (m^5^C) modification is implicated in age‐related diseases, its role in OA remains unclear. Here, the regulatory effects and mechanisms of the m^5^C‐related proteins NSUN2 and ALYREF on chondrocyte senescence and inflammation during OA progression were investigated. NSUN2 expression and m^5^C modification were upregulated in articular chondrocytes from damaged cartilage of surgically induced OA and naturally aged mice, as well as in primary human chondrocyte aging models. NSUN2 was found to promote chondrocyte senescence and NLRP3 inflammasome activation. Messenger RNA (mRNA) m^5^C enrichment, RIP sequencing, and transcriptomic sequencing elucidated the mechanism by which NSUN2 regulates inflammation and aging. IP3R3 was identified as a target gene of NSUN2. Rescue experiments showed that NSUN2 induced Ca^2+^ overload, which was mitigated by 2‐aminoethoxydiphenyl borate (2‐APB) or BAPTA/AM. NSUN2 knockdown or IP3R3 inhibition protected mouse articular cartilage from senescence and NLRP3 inflammasome activation, alleviating OA progression. Mechanistically, NSUN2 cooperated with the m^5^C reader ALYREF to stabilize and promote cytoplasmic export of IP3R3 mRNA, increasing IP3R3 expression. Thus, NSUN2 inhibition reduces chondrocyte senescence and cartilage damage via the IP3R3‐Ca^2+^ axis, may represent a potential therapeutic target for further investigation.

## Graphical abstract

NSUN2‐mediated m^5^C modification cooperates with ALYREF to stabilize and export IP3R3 mRNA, increasing IP3R3 expression and Ca^2^^+^ overload in chondrocytes. This signaling promotes mitochondrial dysfunction, NLRP3 inflammasome activation, and senescence, thereby accelerating osteoarthritis progression.

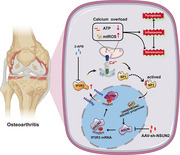

## 1 Introduction

The prevalence of osteoarthritis (OA) with advancing age causes significant pain and disability in patients and a heavy social burden. Aging is the most prevalent factor contributing to OA progression [1, 2]. Cellular senescence and inflammation are crucial indicators of aging that interact with each other [3]. Senescent cells accumulate as an organism ages, thereby decreasing cell proliferation and impairing tissue regeneration and function [4, 5, 6]. Cellular senescence is involved in the pathogenesis and progression of several aging‐related diseases, including OA. Reportedly, inhibiting chondrocyte senescence can delay cartilage aging, thereby preventing and mitigating OA [7, 8]. However, no definitive treatment is available to prevent or treat chondrocyte senescence.

As epigenetic regulators of RNA metabolism, RNA modifications play an important role in the post‐transcriptional modulation of gene expression. Similar to N6‐methyladenosine (m6A), 5‐methylcytosine (m^5^C) is a dynamic and reversible RNA methylation that has recently garnered widespread attention. Initially observed in ribosomal RNA and transfer RNA, m^5^C modification was later discovered in messenger RNA (mRNA) using advanced high‐throughput techniques [9]. m^5^C modification can promote cell senescence, inflammation, and tumor progression [10, 11, 12, 13, 14]. While studies have reported that m6A methylation modification of mRNA can regulate OA development [15, 16, 17], the role of m^5^C methylation in OA is not well studied. Notably, the level of m^5^C modification is higher in advanced OA cartilage than in normal cartilage [18]. However, the relationship between m^5^C methylation and OA development, and the potential of targeting m^5^C modification as a therapeutic strategy for OA, remains insufficiently explored.

Inflammasomes are cytoplasmic multiprotein complexes mediating innate immune responses to microbial infections and cellular injury. Upon activation, the sensor proteins recruit an adaptor apoptosis‐associated speck‐like protein containing CARD (ASC), which subsequently activates caspase‐1 (CASP1). Activated CASP1 promotes the maturation of interleukin (IL)‐1β and IL‐18, and cleaves Gasdermin D (GSDMD), thereby triggering inflammatory signaling and pyroptosis [19, 20]. To date, five pattern‐recognition receptors (PRRs)–NLRP1, NLRP3, NLRC4, Pyrin, and AIM2–have been reported to form inflammasomes [20]. Among them, the NLRP3 inflammasome is the most extensively studied. It can be activated by various danger‐associated signals, including ionic flux, mitochondrial dysfunction, reactive oxygen species (ROS), and mitochondrial DNA (mtDNA) [19, 20, 21]. Reportedly, inflammasome activation is closely associated with cellular senescence and age‐related diseases [22, 23]. Our previous study showed that the NLRP3 inflammasome is significantly activated during OA progression, and targeting this pathway can alleviate chondrocyte senescence and OA severity. However, whether the m^5^C modification contributes to OA progression by regulating inflammasome activation remains unclear.

In the present study, we identified NSUN2 and m^5^C modification as crucial regulators of OA development. Mechanistically, NSUN2, in combination with ALYREF, increases m^5^C modifications and promotes the export and stability of IP3R3 mRNA, thereby increasing NLRP3 inflammasome signaling and promoting chondrocyte senescence by regulating Ca^2+^ flux. The mutation of NSUN2 m^5^C catalytic site or the ALYREF m^5^C recognition site abolished the regulatory effect on the export or stability of IP3R3 mRNA. NSUN2 Knockdown or pharmacological inhibition of IP3R3‐mediated Ca^2+^ flux by 2‐aminoethoxydiphenyl borate (2‐APB) protects chondrocytes against senescence and destabilization of the medial meniscus (DMM) mice against OA.

## 2 Materials and Methods

### 2.1 Chemicals and Reagents

Tert‐butyl hydroperoxide (TBHP) (75‐91‐2) was purchased from Macklin (Shanghai, China), Oridonin (purity > 98%), Z‐DEVD‐fmk (264155), Ac‐YVAD‐cmk (SML0429), LPS (L4391), Adenosine triphosphate (ATP) (A2383‐1G), histamine (H7125) was purchased from Sigma‐Aldrich (St Louis, MO, USA), poly(dA:dT) (tlrl‐patc), was purchased from Invivogen (USA). 2‐APB (524‐95‐8), BAPTA/AM (126150‐97‐8), Plicamycin (18378‐89‐7), 10058‐F4 (403811‐55‐2) were purchased from MCE; NSUN2 (1:10000, 20854‐1‐AP), p21 (28248‐1‐AP, 1:1,000 dilution), TNF‐α (17590‐1‐AP, 1:1000), NLRP3 (68102‐1‐Ig, 1:2,000 dilution), ASC (10500‐1‐AP, 1:5,000 dilution), Caspase1 (22915‐1‐AP, 1:2,000 dilution), IL‐1β (16806‐1‐AP, 1:2,000 dilution), IP3R3 (20729‐1‐AP, 1:1,000 dilution), YBX1 (20339‐1‐AP), GAPDH (HRP‐60004, 1:5,000 dilution) antibodies were purchased from Proteintech (Wuhan, China), COL2A1(GB11021‐100, 1:1000), NEK7 (GB112643‐100, 1:1,000), p16 (GB111605‐100, 1:1,000 dilution) and MMP13 (GB11247‐1‐100, 1:1,000 dilution) antibodies were purchased from Servicebio (Wuhan, China). ALYREF (ab202894, 1:2,000 dilution), m^5^C (ab10805,1:500) were purchased from abcam.

### 2.2 Cell Culture and Treatment

Human cartilage tissues were obtained in compliance with the ethical guidelines approved by the Ethics Committee of the First Affiliated Hospital of Sun Yat‐sen University ([2021]334), with all patients providing informed consent. Cartilage cells were extracted from the tibial plateau and femoral condyle of knee joints from patients undergoing knee replacement surgery for comparative analysis. Cartilage from the relatively intact side was used as the intact group, whereas cartilage from the severely degenerated side was categorized as the osteoarthritis (damaged) group (*n* = 32). The clinical characteristics of OA patients in Table S3. Human primary chondrocytes (HPCs) were isolated according to the protocol described by our previous research [24]. In summary, cartilage samples were minced into small pieces and digested enzymatically with 2 mg/mL collagenase‐II for 12 h at 37°C. The isolated chondrocytes were then cultured in Dulbecco's Modified Eagle Medium (DMEM)/F12, supplemented with 10% Fetal Bovine Serum and 1% penicillin/streptomycin. The cell cultures were maintained at 37°C in a 5% CO_2_ incubator. Chondrocytes were treated with TBHP (100 µM), LPS (100 ng/ml), 2‐APB (50 µM) [21], BAPTA/AM (10 µM) [21], Oridonin (5 µM) [25], histamine (100 µM), Plicamycin (25 nM) or 10058‐F4 (50 µM) for 24 h, Z‐DEVD‐FMK (10 µM), Ac‐YVAD‐cmk (50 µM) [26] was added separately 1 h prior to LPS (100 ng/mL) treatment. Inflammasome‐inducer ATP (5 mM), AIM2 inflammasome‐inducer Poly(dA:dT) (2 µg/ml), or NLRC4 inflammasome‐inducer *Salmonella* (at an MOI of 3) for an additional 1 h after LPS treatment for 24 h.

### 2.3 Western Blot (WB)

The cell pellet was lysed using RIPA lysis buffer (Beyotime) supplemented with phenylmethanesulfonyl fluoride (PMSF, 1 mM, Beyotime) to inhibit protease activity. Protein concentrations were quantified using the bicinchoninic acid (BCA) assay. Protein samples were resolved by sodium dodecyl sulfate‐polyacrylamide gel electrophoresis (SDS‐PAGE) and transferred onto polyvinylidene fluoride (PVDF) membranes. The membranes were pre‐wetted for approximately 10 min using a rapid closure solution before being incubated with the appropriate primary antibodies at 4°C overnight. Following this, the membranes were incubated with their respective horseradish peroxidase (HRP)‐conjugated secondary antibodies for 2 h at room temperature. The immunocomplexes were then detected using a chemiluminescent system with a chemiluminescent substrate (Thermo). Band intensities were quantified using ImageJ software for image analysis.

### 2.4 RNA Extraction and Quantitative Real‐Time PCR (qRT‐PCR) Analysis

Total RNA was extracted from chondrocytes using the RNA‐Quick Purification Kit (ES Science, Shanghai, China) according to the manufacturer's protocol. Complementary DNA (cDNA) was synthesized using the Evo M‐MLV RT Premix (Accurate Biotechnology, Hunan, China). qRT‐PCR was conducted with the SYBR Green Premix Pro Taq HS qPCR kit (Accurate Biotechnology, Hunan, China). Relative gene expression levels were determined employing the 2^−ΔΔCt^ method. The sequences of all primers, sourced from Tsingke Biotechnology (Beijing, China), are detailed in Table S1.

### 2.5 Cells Transfection

Human NSUN2 and ALYREF cDNAs were amplified by PCR and cloned into TK‐PCDH‐copGFP‐T2A‐Puro vectors and the small hairpin RNAs (shRNAs) of NSUN2, ALYREF were synthesized and cloned into the pLKO.1‐CMV‐copGFP‐PURO vector by TsingkeBiotechnologyCo., Ltd. (Beijing, China), Targeted sequences of shRNAs used in this study were listed in Table S2. The Lipofiter 3000 Transfection Reagent (ThermoFisher, China) was used according to the manufacturer's instructions.

### 2.6 β‐Galactosidase Staining

The β‐Galactosidase Staining Kit (Beyotime, Shanghai, China) was utilized to visualize β‐galactosidase activity in cells, following the protocol provided by the manufacturer. Once the staining procedure was complete, images of the stained cells were captured using a standard light microscope.

### 2.7 Cell Activity Assay

The Cell Counting Kit‐8 (CCK‐8) (Beyotime, Shanghai, China) was employed to assess the viability of human chondrocytes. Cells were enumerated and aliquoted into 96‐well plates at a volume of 100 µL per well. Following treatment with the designated reagents, the cells were incubated with the CCK‐8 working solution, diluted to a final concentration of 10%, for a period ranging from 1 to 4 h. The absorbance at 450 nm was then measured using a microplate reader to quantify cell viability.

### 2.8 Transmission Electron Microscopy (TEM)

The cells were fixed using an electron microscope fixative (Servicebio, Wuhan, China) for a duration of 2 h at room temperature. Subsequently, the cells underwent a dehydration process followed by embedding, and then cut into translucent sections. The resulting sections were examined and images were captured using a transmission electron microscope (Hitachi, Tokyo, Japan).

### 2.9 The Intracellular Reactive Oxygen Species (ROS)

1.1. ROS levels were assessed using the ROS assay kit (Beyotime, Shanghai, China), following the manufacturer's instructions. Chondrocytes were incubated with DCFH‐DA for 30 min at 37°C. The images were acquired using fluorescence microscopy.

### 2.10 Measurement of Intracellular Ca^2+^

The fluorescent probe Fluo‐4 AM (S1061S, Beyotime) and Rhod‐2 AM (40776ES50, Yeasen Biotechnology) were used to measure the intracellular Ca^2+^ levels. The processed chondrocytes were preloaded with the Fluo‐4 AM probe (4 µM) to evaluate cytosolic calcium levels ([Ca^2+^]i) or with the Rhod‐2 AM probe (4 µM) in conjunction with MitoTracker Green probe to assess mitochondrial calcium content ([Ca^2+^]m) [27, 28] at 37°C for 30 min in the absence of light. Cell nuclei were stained with Hoechst 33258 dye for 5 min at 37°C. The images were acquired using fluorescence microscopy, and the fluorescence colocalization intensity of MitoTracker and Rhod‐2 AM was used to assess [Ca^2+^]m content.

### 2.11 Measurement of Intracellular ATP

Intracellular ATP levels were assessed using the ATP Assay Kit (S0026, Beyotime, China). The procedure involved lysing whole‐cell extracts from the specified cells using a somatic cellular ATP‐releasing reagent. Following this, the samples were combined with an ATP detection solution, and then quantified using a luminescence plate reader. The results were then normalized against the cellular protein concentration, as determined by the BCA assay.

### 2.12 mRNA Stability Assay

The stably transfected chondrocytes were exposed to 5 µg/mL actinomycin D (ActD) (Selleck, #S8964). Then, the cells were harvested, subsequently, intracellular mRNA was extracted in accordance with the previously outlined protocols. The mRNA levels were normalized to the expression at 0 h.

### 2.13 Enzyme‐Linked Immunosorbent Assay (ELISA)

The supernatants from cell cultures were carefully harvested, and the levels of IL‐1β and IL‐18 were quantified using a specific enzyme‐linked immunosorbent assay (ELISA) kit (MIKX, Shenzhen, China). The assay was performed in accordance with the protocol provided by the manufacturer.

### 2.14 RNA Immunoprecipitation (RIP) and Methylated RNA Immunoprecipitation‐PCR (MeRIP‐qPCR) Analysis

The RIP experiment was carried out with PureBinding RNA Immunoprecipitation Kit (Guangzhou, China, Geneseed, P0101) according to the manufacturer's instructions. Magnetic beads were mixed with 5 µg of anti‐NSUN2 (Abcam, ab259941) or anti‐rabbit IgG (Abcam, ab172730), the treated magnetic beads were added to chondrocytes lysates, and the mixtures were incubated at 4°C for 12 h. Next, proteinase K digestion buffer was added to digest the complexes for 45 min at 55°C. After that, buffer (phenol:chloroform:isoamyl alcohol = 125:24:1) was employed to extract the RNA. The extracted RNA was subsequently analyzed by qRT–PCR. IgG was used as a negative control to preclude nonspecific binding. Similar to that for RIP, a m^5^C MeRIP kit (BersinBio, Guangzhou, China; Bes5204‐2) was used according to the manufacturer's instructions. In short, total RNA was isolated then 1/10 volume of fragmented RNA sample was saved as “Input”. After incubated with 5ug of anti‐m5 C antibody, or anti‐IgG at 4°C for 4 h, the complexes were digested by proteinase K digestion buffer. RNA was purified and analyzed by qRT‐PCR with normalization to the input.

### 2.15 RNA‐Sequencing (RNA‐Seq) and m^5^C MeRIP‐Sequencing (MeRIP‐Seq)

For RNA‐Seq, total RNA from each sample was extracted using Invitrogen TRIzol Reagent, the libraries were then analyzed using the Agilent Bioanalyzer 2100, quantified via qRT‐PCR, equal amounts of RNA were pooled for RNA‐Seq library construction. Construction of the RNA‐seq library and RNA‐seq was performed by Tsingke Biotechnology (Beijing, China). The m^5^C MeRIP‐Seq service was provided by CloudSeq Inc. (Shanghai, China). Total RNA was subjected to immunoprecipitation with the GenSeq m^5^C MeRIP Kit (GenSeq Inc.) by following the manufacturer's instructions. Briefly, after coimmunoprecipitating with specific m^5^C antibodies. RNA libraries for IP and input samples were then constructed with GenSeq Low Input Whole RNA Library Prep Kit (GenSeq, Inc.) by following the manufacturer's instructions. Libraries were qualified using Agilent 2100 bioanalyzer (Agilent) and then sequenced.

### 2.16 DNA Damage Assay Kit by γH2AX Immunofluorescence

DNA Damage was detected by DNA Damage Assay Kit by γH2AX Immunofluorescence (Beyotime, C2035S) according to the manufacturer's instructions. Then the images were acquired using fluorescence microscopy.

### 2.17 Dot Blot Assay

The RNA was combined with denaturation buffer and incubated at 95°C for 5 min, followed by immediate cooling on ice. Subsequently, an equal volume of precooled 20× SSC was added to the RNA sample and thoroughly mixed. The RNA was then applied to an nitrocellulose filter membrane (Servicebio, G6014) and fixed by crosslinking with UV light. The membrane was prepped with a 5% nonfat milk solution in PBST for 1 h at room temperature. It was subsequently incubated with a primary anti‐m^5^C antibody overnight at 4°C. After thorough washing with PBST, the membrane was treated with a HRP‐conjugated secondary antibody for 1 h and visualized using a chemiluminescence imaging system. The membrane were then stained with methylene blue.

### 2.18 Immunofluorescence (IF) and RNA Fluorescence In Situ Hybridization (RNA‐FISH)

Cells were permeabilized using 0.5% Triton X‐100 for 20 min, then blocked with 1% goat serum albumin for 1 h at 37°C. They were then incubated with primary antibodies specific for NSUN2, NEK7 or NLRP3 overnight at 4°C. The cells were further incubated with fluorophore‐conjugated secondary antibodies for 1 h, after DAPI was added to the cells, and images were acquired with a fluorescence microscope. For RNA‐FISH combined with IF staining, chondrocytes were fixed with 4% paraformaldehyde. After 1 h of prehybridization, overnight hybridization was performed at 42°C using specific probes. Cell nuclei were stained with 2 mg/mL of DAPI for 8 min at 20°C–25°C. Images were acquired using a confocal microscope (Zeiss LSM710). Probes of IP3R3 transcripts are as follows: GTTCTTGTAGTCCCACTGGTTGGCG.

### 2.19 Animal Experiment

Male C57BL/6 mice (8 weeks old) were obtained from Guangdong Medical Laboratory Animal Center (Guangdong, China), and animal use and animal protocols have been approved by Institutional Animal Care and Use Committee of Sun Yat‐Sen University (SYSU‐IACUC‐2024001532). Animal experiments were conducted in compliance with the Basel Declaration. Surgical destabilization of the medial meniscus (DMM) was used to induce OA in the left hind knee of mice, and the procedure was performed in accordance with previous reports [29, 30]. Five mice were randomly assigned to the sham group and underwent arthrotomy in the left hind knee as control group, then 20 mice were randomly divided into the following four groups (*n* = 5): DMM + AAV‐sh‐Control (sh‐Control) group, DMM + AAV‐sh‐NSUN2 group (sh‐NSUN2) and DMM + DMSO group (DMSO), DMM + 2‐APB group (2‐APB). At eight weeks post‐surgical induction, all mice were sacrificed and tissues were harvested for biochemical and histological analyses. AAV‐sh‐NSUN2 or AAV‐sh‐Control was purchased from Hanbio Tech (Shanghai, China). Virus in a total volume of 10 µl was injected into the knee joints of mice at 1 week after DMM surgery. 2‐APB or an equal volume of DMSO was administered to mice at a concentration of 2 mg/kg via intraperitoneal injection on the second day after DMM surgery, once every other day for a duration of six weeks [30].

### 2.20 Histological and Immunohistochemical Analyses

Mice knee joint tissues or human OA knee cartilage tissue were fixed in a 4% paraformaldehyde solution overnight, followed by embedding in paraffin. The embedded tissues were sectioned into 5‐micrometer‐thick slices. These sections underwent deparaffinization in xylene and were rehydrated using a graded ethanol series in water. The samples were then treated with 3% hydrogen peroxide (H_2_O_2_) to quench endogenous peroxidase activity and subsequently blocked with goat serum. The sections were incubated with primary antibodies of NSUN2, IP3R3, IL‐1β, MMP13 or stained with hematoxylin‐eosin (HE) and Safranine O (SO) at 4°C overnight. After washing, they were incubated with the appropriate secondary antibodies. Then the stained sections were examined under a microscope. Histopathological analysis was conducted using ImageJ software, and the severity of OA was evaluated according to the Osteoarthritis Research Society International (OARSI) histopathology scoring system.

### 2.21 Micro‐CT

The knee joints of the mice were fixed in a 4% paraformaldehyde solution and subjected to microstructural analysis using a micro‐CT scanner (Bruker SkyScan1276, Bruker, Belgium). Scanning was performed at a tube voltage of 70 kV and a tube current of 200 µA, with an integration time of 460 ms and an isotropic voxel size of 20.07 µm. Subsequently, three‐dimensional reconstructions of the joint structures were generated using Mimics software.

### 2.22 Statistical Methods

All experiments were conducted a minimum of three times, with results presented as the mean ± standard deviation. Statistical evaluations were carried out using a one‐way analysis of variance (ANOVA) for parametric data, and the Student's t‐test and Kruskal‐Wallis H test for non‐parametric data (OARSI score and synovitis scores). A p‐value of less than 0.05 was regarded as indicative of statistical significance. Data visualization was accomplished using GraphPad Prism software, version 8.0.2.

## 3 Results

### 3.1 m^5^C Modification Levels and NSUN2 Expression are Significantly Increased in Senescent Chondrocytes and OA Cartilage

**FIGURE 1:**
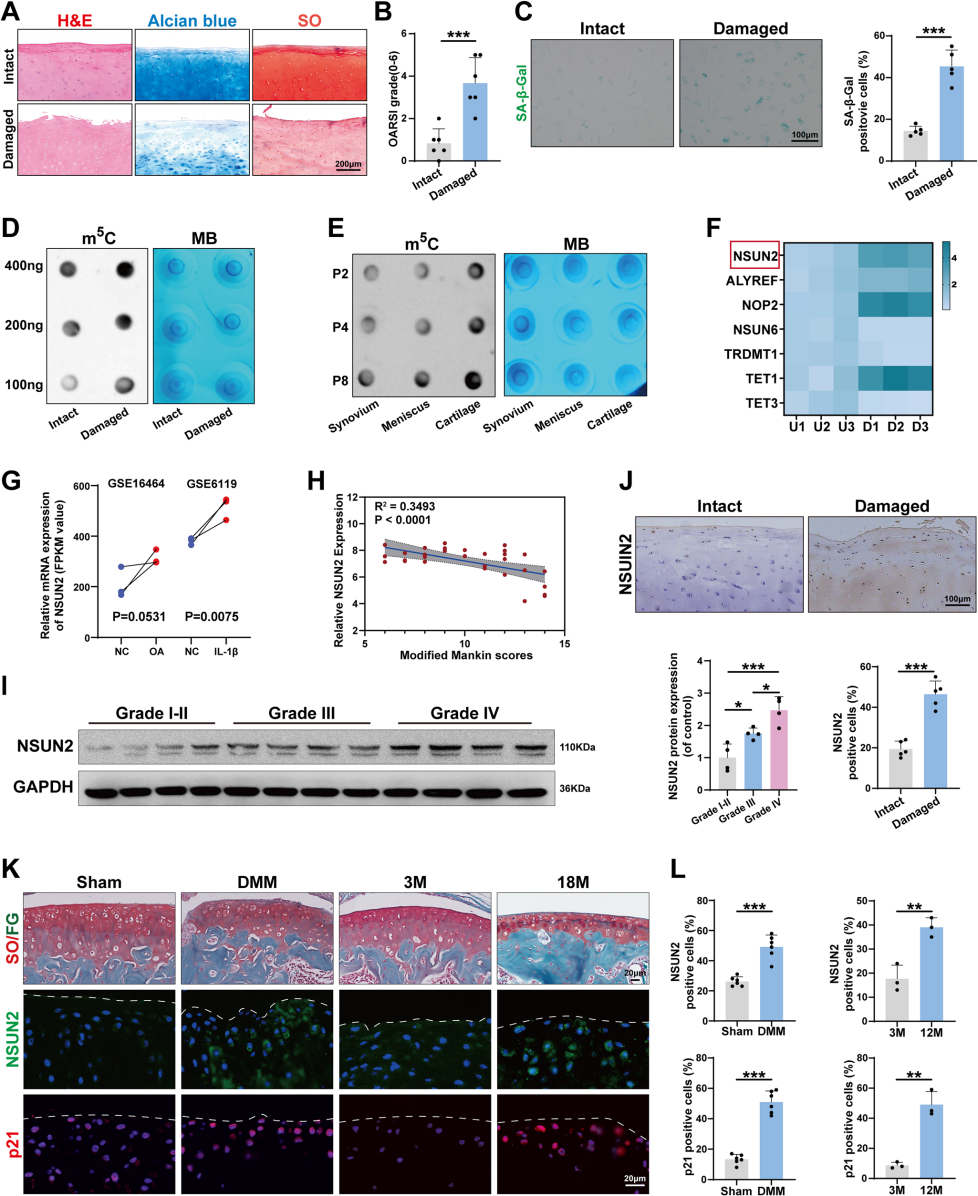
m^5^C level and methyltransferase NSUN2 expression are increased in senescent chondrocytes and OA cartilage tissue. (A, B) Representative images of Hematoxylin and Eosin staining (HE), Alcian blue and Safranin O staining (SO) and quantification of OARSI in intact and damaged articular cartilage sections collected from OA patients. N = 6, bars = 200 µm. (C) Images and quantification of SA‐β‐Gal positivity in intact and damaged articular cartilage sections collected from OA patients. N = 5, bars = 100 µm. (D, E) m^5^C Dotblot analysis of the m^5^C modification levels of mRNA extracted from chondrocytes of intact and damaged articular cartilage and different tissue cells in knee joint at passage 2,4 and 8. N = 3. (F) RNA‐seq showing the expression of genes associated with m^5^C modification in intact and damaged articular cartilage sections collected from OA patients. (G) The expression of NSUN2 in human chodrocytes extracted from normal vs OA articular cartilage from GSE16464, and human primary chondrocytes treated with IL‐1β vs control from GSE 6119. (H) The correlations between NSUN2 and a modified Mankin grade were validated. N = 32 independent biological replicates. (I) The association between NSUN2 and OA severity was detected by western blot. N = 4 independent biological replicates per group. (J) Representative images and quantification of immunohistochemistry staining of NSUN2 in articular cartilage of intact and damaged articular cartilage. N = 5, bars = 100 µm. (K, L) Representative images of Safranin O/Fast Green staining and immunofluorescence of NSUN2 and p21 and quantification of NSUN2‐, p21‐positive chondrocytes in articular cartilage from mice of control versus 8 weeks post DMM surgery (N = 5) and aged 3 months versus 18 months (N = 3), bars = 20 µm. (Data was presented as mean ± SD, *** *p* < 0.001, ** *p* < 0.01, * *p* < 0.05).

To investigate the association between m^5^C modification and cellular senescence during OA progression, we evaluated m^5^C modification levels in damaged and intact cartilage obtained from patients who underwent total knee replacement (Figure 1A,B). Consistent with previous studies, damaged cartilage showed considerably increased SA‐β‐gal positivity, indicating more pronounced senescence phenotypes (Figure 1C). Anti‐m^5^C dot blot assays showed that mRNA m^5^C modification levels were significantly higher in damaged cartilage than in intact cartilage (Figure 1D). We assessed m^5^C levels in senescent HPC models (P8‐ and TBHP‐treated), which exhibited increased cellular senescence and OA phenotypes, including increased SA‐β‐gal activity and upregulated p16INK4a, p21, TNF‐α, and MMP13 expression, but decreased COL2A1 (Figure S1A–D). Higher m^5^C modification levels with greater variation between passages were observed in chondrocytes than in synovial and meniscal cells (Figure 1E).

To further evaluate m^5^C‐related enzyme levels, we screened for differentially expressed mRNAs of m^5^C‐associated genes using RNA sequencing data from HPCs isolated from damaged and intact cartilage of patients with OA. Results showed that differential expression of NSUN2, ALYREF, NOP2, NSUN6, TRDEMT1, TET1, and TET3 mRNA. Among these, the m^5^C methyltransferase NSUN2 was significantly upregulated (Figure 1F), which was confirmed by external RNA‐seq datasets (GSE16464: Control vs. OA; GSE6119: Control vs. IL‐1β‐treated; Figure 1G). Analysis of human cartilage tissues revealed increased NSUN2 mRNA and protein levels in damaged cartilage, which positively correlated with OA severity (Figure 1H,I). IHC analysis confirmed an increase in NSUN2‐positive chondrocytes in the damaged regions (Figure 1J). NSUN2 expression was upregulated in TBHP‐treated chondrocytes and late‐passage cells (Figure S1E–H). The OA severity correlates with upregulation expression of p21 and NSUN2 in DMM mice model (Figure 1K,L). NSUN2 expression was also higher in the knee cartilage of aged (18‐month‐old) mice than in that of young (3‐month‐old) mice (Figure 1K,L). These results reveal increased m^5^C levels and NSUN2 expression in senescent chondrocytes and OA cartilage, suggesting the role of mRNA m^5^C modification in cartilage aging and OA progression.

### 3.2 NSUN2 Promotes Chondrocyte Senescence by Regulating m^5^C Modification

**FIGURE 2:**
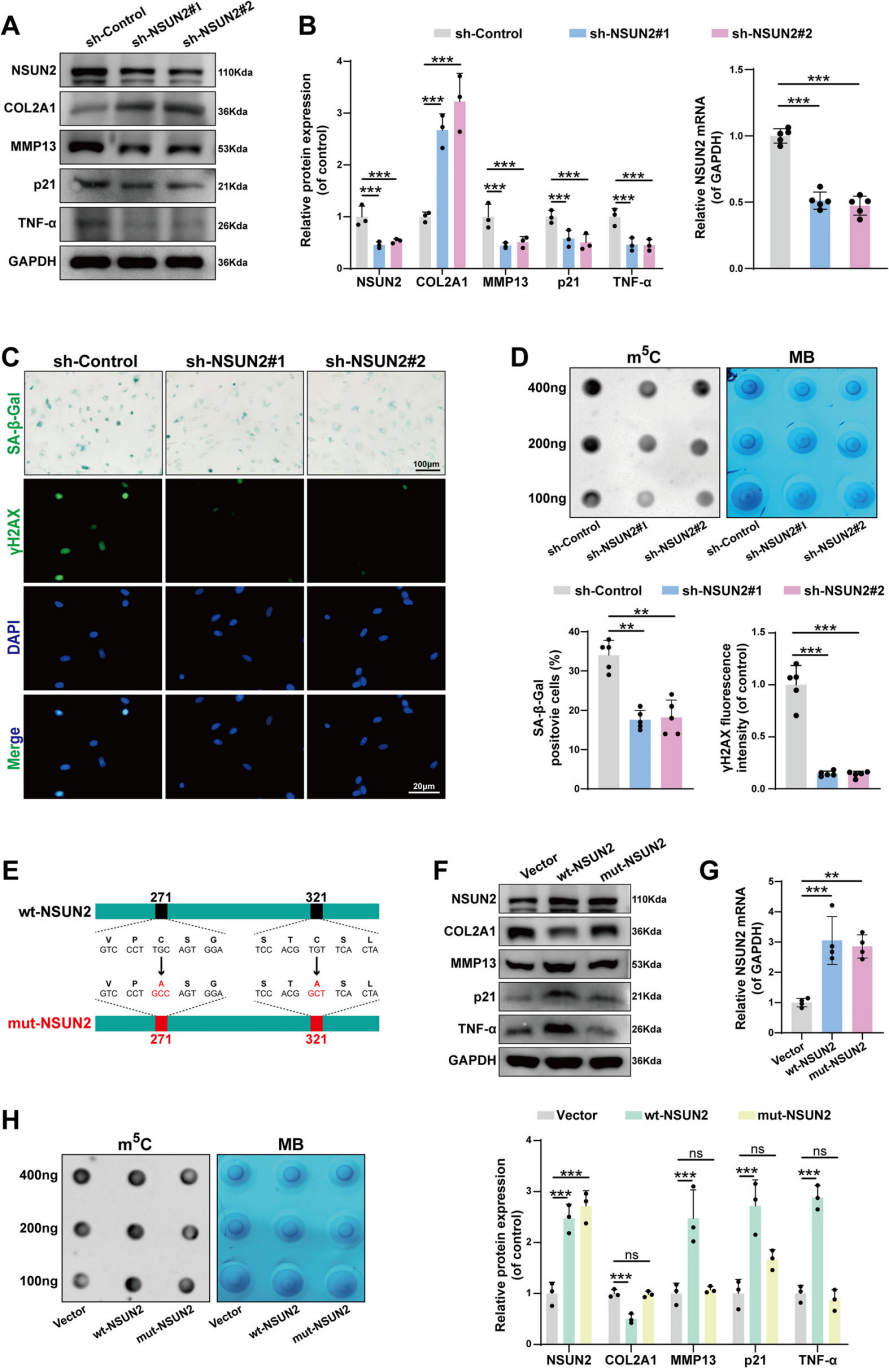
NSUN2 positively regulates m^5^C and promote cellular senescence by m^5^C in chondrocytes (A, B) Relative mRNA of NSUN2 and protein expression level of NSUN2, COL2A1, MMP13, p21, and TNF‐α in primary human chondrocytes (HPCs) at passage 8 transfected with shRNA targeting NSUN2 (sh‐NSUN2) compared to negative control shRNA (sh‐Control). (C) Images and quantification of SA‐β‐Gal and IF staining of γH2AX in HPCs transfected with sh‐Control, sh‐NSUN2#1, sh‐NSUN2#2. N = 5, bars = 100 (SA‐β‐Gal) or 20 µm (γH2AX). (D) Dotblot analysis of the m^5^C modification levels of mRNA in HPCs transfected with sh‐Control, sh‐NSUN2#1, sh‐NSUN2#2. (E) Schematic representation of the two sites of NSUN2 mutated to alanine. (F, G) Relative mRNA of NSUN2 and protein expression level of NSUN2, COL2A1, MMP13, p21, and TNF‐α in HPCs at passage 2 transfected with plasmid encoding wildtype NSUN2 (wt‐NSUN2) or mutant NSUN2 (mut‐NSUN2) or plasmid control with no insert (Vector). (H) Dotblot analysis and quantification of the m^5^C modification levels of mRNA of HPCs transfected with Vector, wt‐NSUN2, mut‐NSUN2. (Data was presented as mean ± SD, ^***^ *p* < 0.001, ^**^ *p* < 0.01, ns, no significance).

To explore the role of NSUN2 in chondrocyte senescence during OA progression, we knocked down NSUN2 using shRNA (Figure 2A,B). NSUN2 knockdown significantly reduced the ratio of SA‐β‐Gal‐positive cells and decreased DNA damage, senescence markers (p21), and SASP factors (TNF‐α and MMP13), while upregulating COL2A1 expression (Figure 2A–C). Anti‐m^5^C dot blot assays showed that NSUN2 knockdown considerably decreased mRNA m^5^C modification levels (Figure 2D), confirming that NSUN2 is the primary regulator of m^5^C modification in chondrocytes. These results suggest that NSUN2 promotes chondrocyte senescence and mediates mRNA m^5^C modification.

To determine whether NSUN2 regulates chondrocyte senescence via m^5^C methyltransferase activity, we constructed an enzymatically inactive NSUN2 double‐site mutant (C271A/C321A) based on the published catalytic sites (Figure 2E) [9]. Overexpression of wild‐type NSUN2 (wt‐NSUN2) or mutant NSUN2 (mut‐NSUN2) in chondrocytes significantly increased NSUN2 mRNA and protein levels (Figure 2F,G). However, only wt‐NSUN2 overexpression increased mRNA m^5^C modification levels, whereas mut‐NSUN2 exhibited no effect (Figure 2H), confirming the successful ablation of its catalytic activity. Furthermore, overexpression of wt‐NSUN2, but not mut‐NSUN2, promotes chondrocyte senescence, as indicated by increased SA‐β‐Gal positivity, DNA damage, and senescence‐associated protein expression (Figure 2F,G, Figure S2A,B). These results suggest that NSUN2 promotes chondrocyte senescence via its m^5^C methyltransferase activity.

### 3.3 NSUN2 Promotes Activation of the NLRP3 Inflammasome Pathway

**FIGURE 3:**
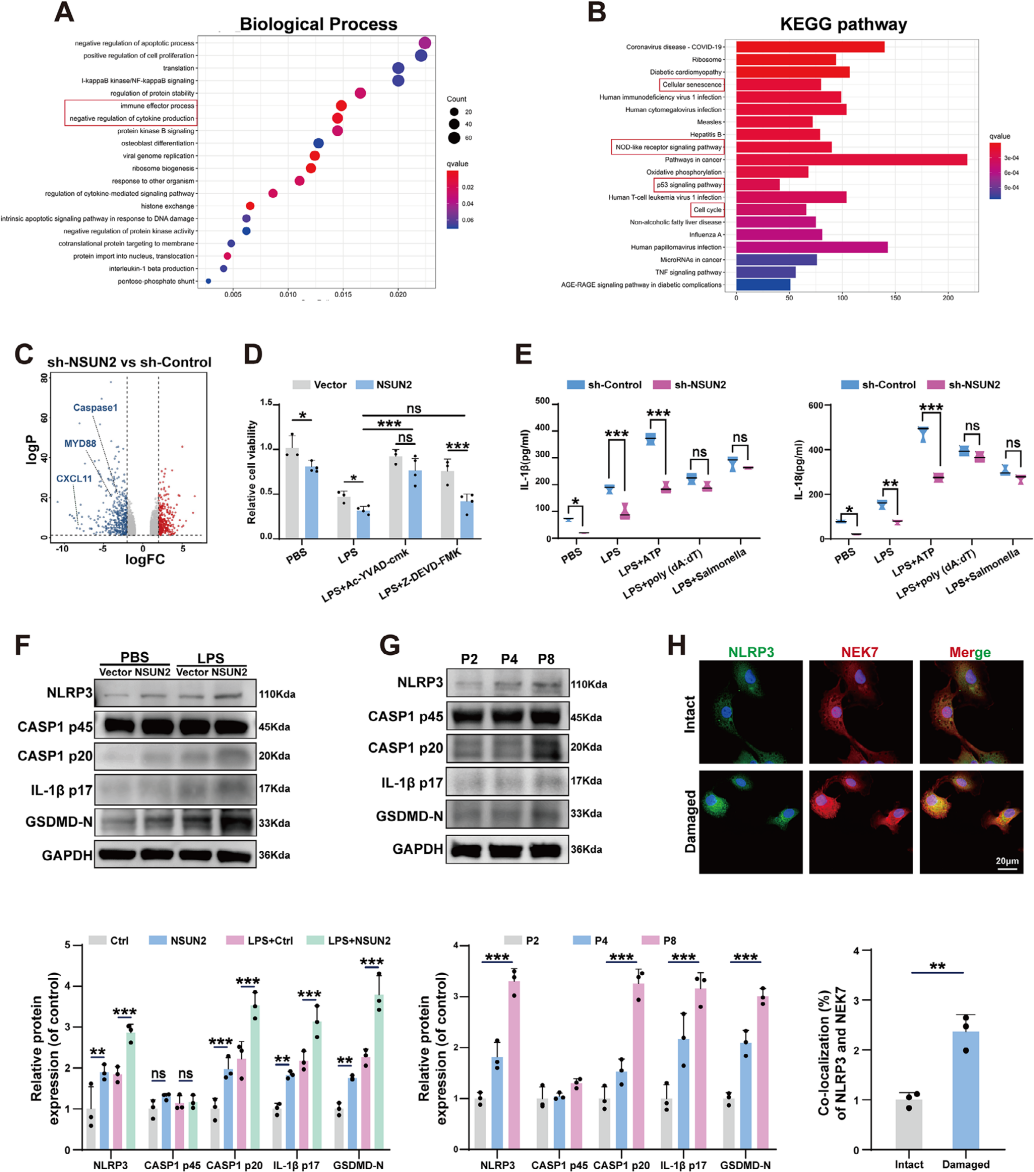
NSUN2 promotes the activation of NLRP3 inflammasome pathway in chondrocytes. (A–C) RNA‐seq with human primary chondrocytes (HPCs) stably transfected with sh‐NSUN2 or sh‐Control. (A) GO analysis of upregulated genes for biological processes. (B) Pathway enrichment analysis of differentially expressed genes. (C) Volcano plot demonstrating differentially expressed genes (fold change >2 or <0.5). (D) HPCs stably transfected with oe‐NSUN2 (NSUN2) or oe‐Control (Vector) then CCK‐8 assay was performed to determine the cell viability after treatment with PBS, LPS, apoptosis inhibitor Z‐DEVD‐fmk and pyroptosis inhibitor Ac‐YVAD‐cmk in the indicated cells. (E) ELISA analysis of IL‐1β, and IL‐18 in supernatants of chondrocytes stably transfected with sh‐NSUN2 or sh‐Control following priming with LPS (100 ng/mL) for 24 h and subsequent stimulation with ATP, or poly (dA:dT) or salmonella for 1 h. (F) Inflammasome‐related protein expression level and quantification in HPCs treatment with PBS or LPS, following transfection with NSUN2 or Vector. (G) The protein expression levels and quantification of different Inflammasome‐related proteins in HPCs at different passage. (H) Representative IF images of NLRP3 and NEK7 in chondrocytes of intact and damaged articular cartilage. N = 3, bars = 20 µm. (Data was presented as mean ± SD, ^***^ *p* < 0.001, ^**^ *p* < 0.01, ^*^ *p* < 0.05, ns, no significance).

To elucidate the molecular mechanisms underlying NSUN2‐mediated chondrocyte dysfunction, we performed RNA‐seq analysis using primary OA chondrocytes stably transfected with sh‐NSUN2#1 or sh‐control. GO biological process analysis showed a significant enrichment of pathways related to immune effector processes and cytokine regulation (Figure 3A). KEGG pathway analysis identified the enrichment of the senescence pathway, NOD‐like receptor signaling pathway, p53 signaling, and cell cycle regulation (Figure 3B). Several genes associated with inflammatory signaling, including Caspase1, MYD88, and CXCL11 were significantly downregulated after NSUN2 knockdown (Figure 3C). These findings suggest that in addition to regulating senescence‐associated phenotypes NSUN2 may also participate in inflammatory signaling pathways.

Therefore, we focused on inflammasome activation. The NLR signaling pathway is an important innate immune defense mechanism that recognizes diverse danger signals, including pathogens, cellular damage, and autonucleic acids, through components such as PRRs, inflammasomes, caspase‐1, and related inflammatory factors. However, overactivation can lead to pyroptosis [19, 20]. Previous studies have implicated inflammasomes in promoting cell senescence and OA progression [22, 23, 24]. To investigate whether NSUN2 regulates chondrocyte inflammasomes, we treated chondrocytes with the apoptosis inhibitor Z‐DEVD‐FMK or the pyroptosis inhibitor Ac‐YVAD‐cmk. CCK‐8 assays revealed that NSUN2 overexpression reduced chondrocyte viability regardless of LPS stimulation. This reduction was rescued only by Ac‐YVAD‐cmk, not by Z‐DEVD‐FMK (Figure 3D).

Because inflammasomes include several types, including NLRP1, NLRP3, AIM2, and NLRC4, we further examined which specific inflammasome was regulated by NSUN2. LPS‐primed chondrocytes were stimulated with the NLRP3 inflammasome activator ATP, the AIM2 inflammasome activator poly(dA:dT), or the NLRC4 inflammasome inducer Salmonella. ELISA showed that NSUN2 deficiency considerably suppressed IL‐1β and IL‐18 secretion induced by NLRP3 inflammasome activation, but not by NLRC4 or AIM2 inflammasome activation (Figure 3E). These results reveal a selective role for NSUN2 in regulating the NLRP3 inflammasome, which was further supported by increased protein levels of NLRP3, CASP1 p20, IL‐1β p17, and GSDMD‐N upon NSUN2 expression (Figure 3F).

We investigated whether inflammasome activation is associated with chondrocyte aging during OA progression. NLRP3 expression and inflammasome‐related markers were increased in senescent chondrocytes (Figure 3G). Immunofluorescence analysis showed increased colocalization of NLRP3 and NEK7 in chondrocytes derived from damaged OA cartilage (Figure 3H), indicating increased inflammasome assembly/activation in the degenerative cartilage. Altogether, these results suggest that NSUN2 contributes to OA progression by promoting NLRP3 inflammasome activation.

### 3.4 NSUN2 Stimulates IP3R3 Expression in Chondrocytes

**FIGURE 4:**
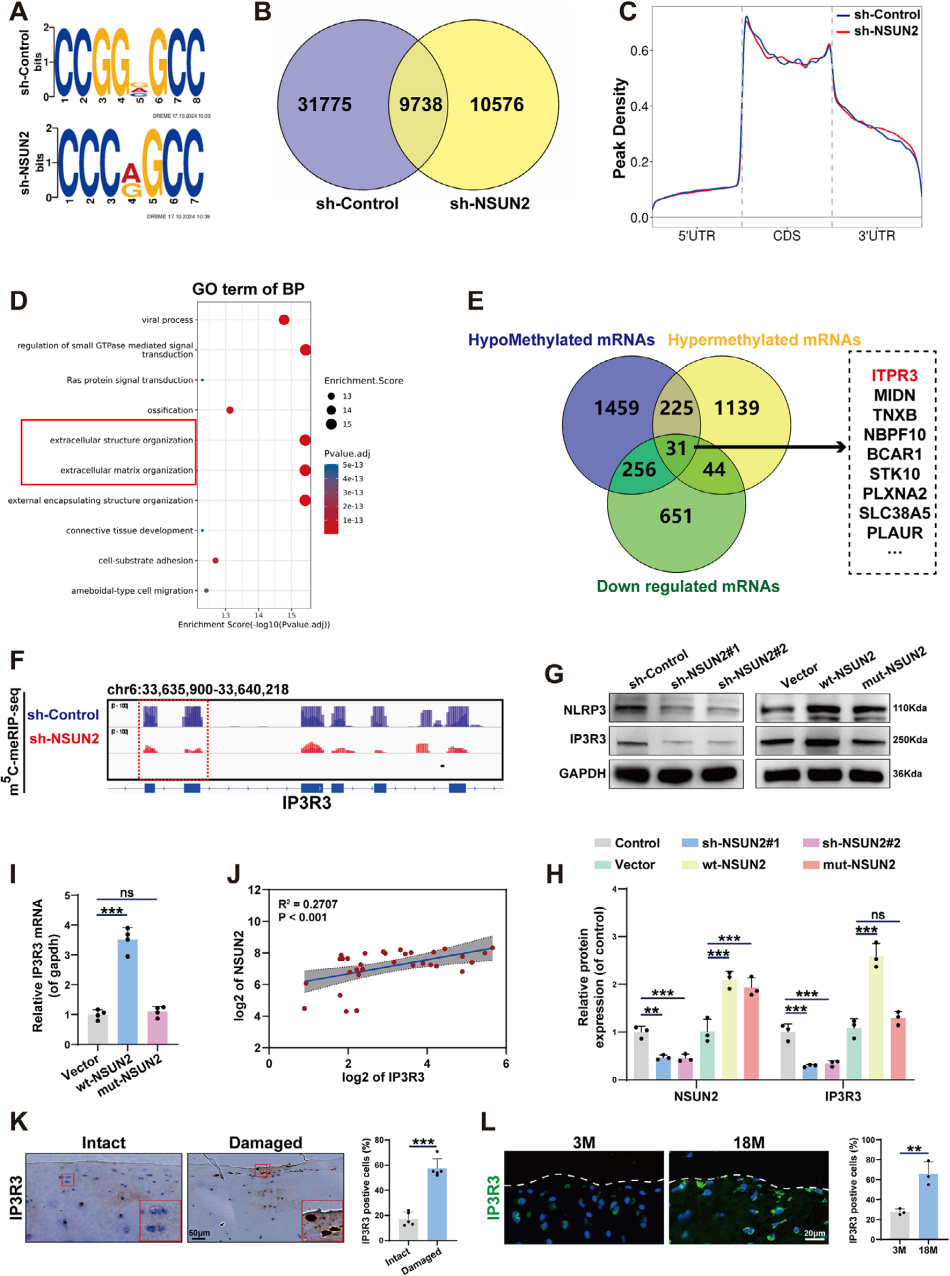
IP3R3 is the potential target of NSUN2 in chondrocytes. (A) The top consensus m^5^C motif in in sh‐NSUN2 and control human primary chondrocytes (HPCs). (B) The peak denisity of m^5^C in sh‐NSUN2 and sh‐Control HPCs. (C) The m^5^C distributions within different regions in sh‐NSUN2 and sh‐Control HPCs. (D) GO analysis of hypomethylated genes for biological processes compared sh‐NSUN2 with sh‐Control HPCs. (E) Venn diagram illustrating the intersection of significantly hypomethylated and downregulated mRNAs following NSUN2 knockdown in HPCs, and hypermethylated mRNAs in OA HPCs. ITPR3 is the most significantly differential gene in the intersection. (F) IGV tracks from m^5^C‐meRIP‐seq analysis showing m^5^C enrichment of IP3R3. (G, H) Relative protein of IP3R3 when NSUN2 was knocked down and wt‐NSUN2 or mut‐NSUN2 was overexpressed in HPCs. (I) Relative mRNA of IP3R3 when wt‐NSUN2 or mut‐NSUN2 was overexpressed in HPCs. (J) Pearson correlation analysis showing the association between NSUN2 and IP3R3 mRNA expression in HPCs. N = 32. (K, L) Representative images and quantification of immunohistochemistry staining of IP3R3 in intact or damaged articular cartilage (N = 5, bars = 50 µm) and in articular cartilage of mice aged 3 months or 18 months using IF (N = 3, bars = 20 µm) (Data was presented as mean ± SD, ^***^ *p* < 0.001, ^**^ *p* < 0.01, ns, no significance).

To identify potential targets through which NSUN2 regulates inflammasome activation and senescence in chondrocytes, we performed m^5^C MeRIP‐seq analysis using HPCs stably transfected with sh‐NSUN2#1 or sh‐control. Consistent with previous findings, the top consensus m^5^C motif in sh‐NSUN2 and control chondrocytes was enriched in CG‐rich sequences (Figure 4A) [9]. The m^5^C peaks were located in coding sequence regions, with a significant reduction in peak number upon NSUN2 knockdown (Figure 4B,C). Transcripts identified by MeRIP‐seq were enriched in OA‐associated signaling pathways, including extracellular matrix organization and extracellular structure organization (Figure 4D). Through integrated analysis of our RNA‐seq (|log2FC| ≥ 1) and MeRIP‐seq (FC ≥ 100) data with published MeRIP‐seq data (FC ≥ 100) from OA and control chondrocytes [18], we identified 31 significantly altered genes. ITPR3 (encoding inositol 1,4,5‐trisphosphate receptor type 3; IP3R3) showed the most pronounced difference (Figure 4E). Furthermore, RNA sequencing revealed significantly higher IP3R3 expression in chondrocytes from damaged and intact cartilages (Figure S3A). IGV visualization confirmed the reduced m^5^C peaks within ITPR3 upon NSUN2 knockdown (Figure 4F). IP3R3 levels decreased following NSUN2 knockdown but increased specifically upon wt‐NSUN2 overexpression, but not upon mut‐NSUN2 overexpression (Figure 4G). IP3R3 mRNA levels (but not IP3R1 or IP3R2) increased only with wt‐NSUN2 overexpression (Figure 4I, Figure S3B). IP3R3 mRNA levels positively correlated with NSUN2 mRNA levels (Figure 4J). Altogether, these results show that NSUN2 regulates IP3R3 expression at both the mRNA and protein levels.

We validated IP3R3 expression during OA progression in vitro and in vivo. WB revealed significantly higher IP3R3 levels in human chondrocytes after serial passaging or TBHP treatment (Figure S3C,D). IHC staining showed significantly higher IP3R3 levels in damaged cartilages than in intact cartilages (Figure 4K). We confirmed that IP3R3 expression was upregulated in the knee cartilage of 18‐month‐old mice compared with 3‐month‐old mice (Figure 4L). These results suggest that IP3R3 is highly expressed in aged cartilage, supporting its role as a target gene of NSUN2 in regulating age‐related OA progression.

### 3.5 NSUN2 Mediates the m^5^C‐Dependent IP3R3 mRNA Export and Stability in an ALYREF‐Dependent Manner

**FIGURE 5:**
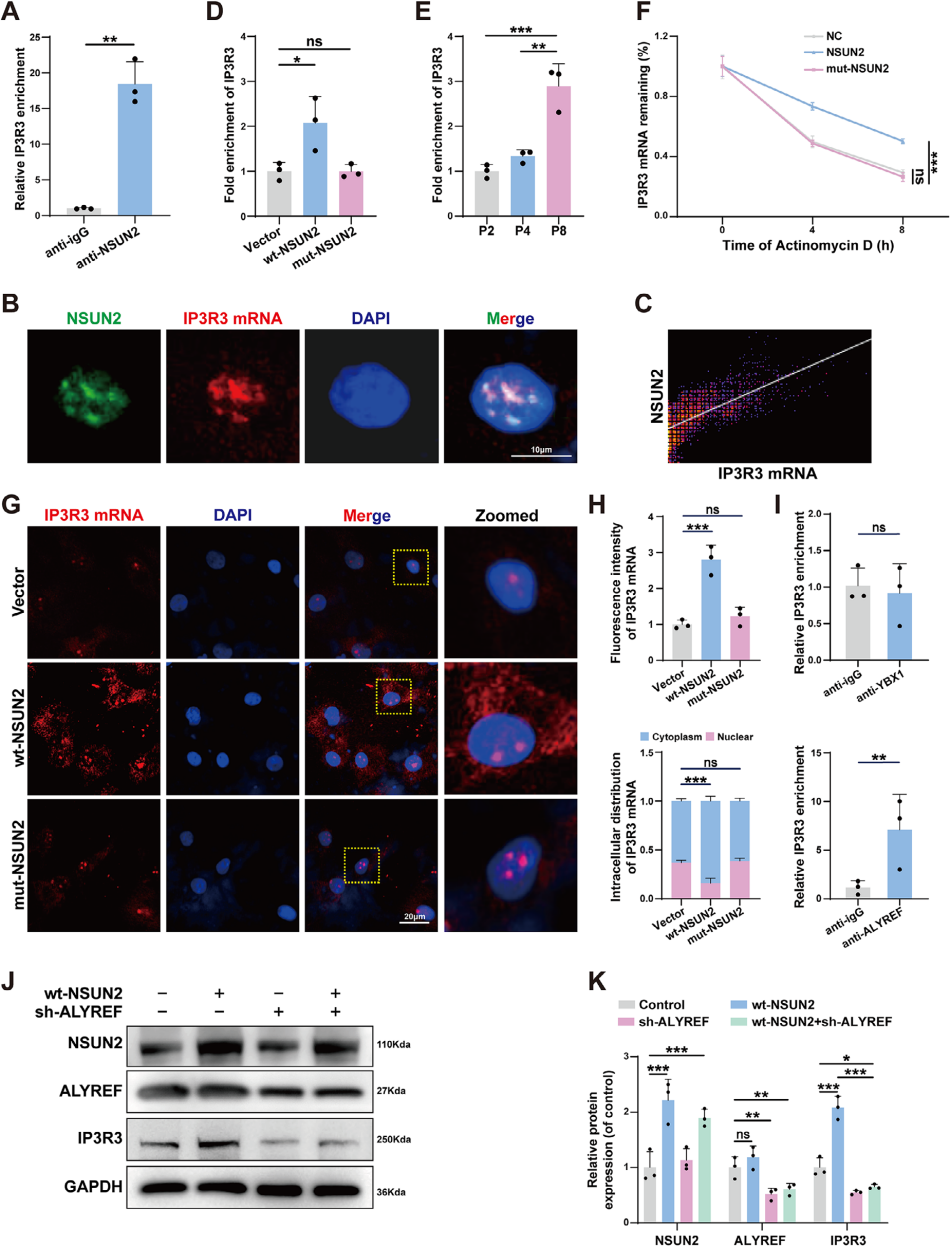
NSUN2 promotes the export and enhances the stability of IP3R3 mRNA by m^5^C modification in an ALYREF‐dependent manner. (A) RIP/RT‐qPCR assays showing the binding of NSUN2 to IP3R3 mRNAs in primary human chondrocytes (HPCs). N = 3. (B, C) Representative fluorescence in situ hybridization (FISH) and Immunofluorescence images and quantifications showed that the co‐localization of NSUN2 and IP3R3 mRNA. N = 3, bars = 10 µm. (D, E) The relative m^5^C enrichment of IP3R3 mRNA in HPCs transfected with different plasmids, and different passages, each group was normalized to the Input. (F) RNA stability assay showing IP3R3 mRNA half‐life in HPCs transfected with wt‐NSUN2 or mut‐NSUN2 plasmids N = 3. (G, H) Representative FISH images and quantifications showed that the distribution of IP3R3 mRNA in HPCs. N = 3, bars = 20 µm. (I) RIP /RT‐qPCR assays showing the binding of ALYREF or YBX1 to IP3R3 mRNAs. N = 3. (J, K) Relative protein expression level of NSUN2, ALYREF and IP3R3 in HPCs transfected with plasmid encoding wt‐NSUN2 or sh‐ALYREF. (Data was presented as mean ± SD, ^***^ *p* < 0.001, ^**^ *p* < 0.01, ^*^ *p* < 0.05, ns, no significance).

We investigated whether NSUN2's regulation of IP3R3 expression depends on its m^5^C methyltransferase activity. RIP analysis showed significant enrichment of IP3R3 mRNA in NSUN2 immunoprecipitates than in IgG controls (Figure 5A). FISH combined with IF imaging showed substantial colocalization of NSUN2 protein and IP3R3 mRNA in HPCs (Figure 5B,C). m^5^C MeRIP‐qPCR assays showed that overexpression of wt‐NSUN2, but not its catalytically inactive mutant, significantly increased the m^5^C modification levels of IP3R3 mRNA in HPCs (Figure 5D). The IP3R3 mRNA m^5^C levels increased during HPC passage (Figure 5E). Based on the roles of m^5^C modifications in mRNA metabolism, including maintenance of RNA stability and nuclear export [9, 10, 11], we examined whether NSUN2 affects IP3R3 mRNA stability or export. Actinomycin D experiments showed that overexpression of wt‐NSUN2, but not the mutant, stabilized IP3R3 mRNA (Figure 5F). FISH analysis showed that wt‐NSUN2 overexpression, in contrast to the mutant, increased the nuclear export of IP3R3 mRNA (Figure 5G,H).

Because ALYREF and YBX1 are widely recognized m^5^C reader proteins [9, 11], we performed RIP assays to determine their physical interaction with IP3R3 mRNA. We found a significant enrichment of IP3R3 mRNA in ALYREF immunoprecipitates, consistent with the ALYREF RIP‐Seq findings in HeLa cells [9]. However, no enrichment was observed in the YBX1 immunoprecipitates (Figure 5I). Because ALYREF promotes mRNA export in HeLa cells [9], we investigated whether it exerts a similar function in chondrocytes. Overexpression of wild‐type ALYREF (wt‐ALYREF) increased IP3R3 mRNA and protein levels. However, the overexpression of the K171A catalytic mutant ALYREF (mut‐ALYREF) [9] exerted no significant effect (Figure S4A–C). Consistently, wt‐ALYREF overexpression, but not mut‐ALYREF overexpression, increased the nuclear export and stabilized IP3R3 mRNA (Figure S4D–F). ALYREF knockdown attenuated the increase in IP3R3 levels induced by NSUN2 overexpression (Figure 5J,K). Altogether, these findings indicate that NSUN2 upregulates IP3R3 expression by promoting the export and stability of IP3R3 mRNA via m^5^C modification in an ALYREF‐dependent manner.

### 3.6 NSUN2 Regulates Ca^2+^ Flux by IP3R3 in Chondrocytes

**FIGURE 6:**
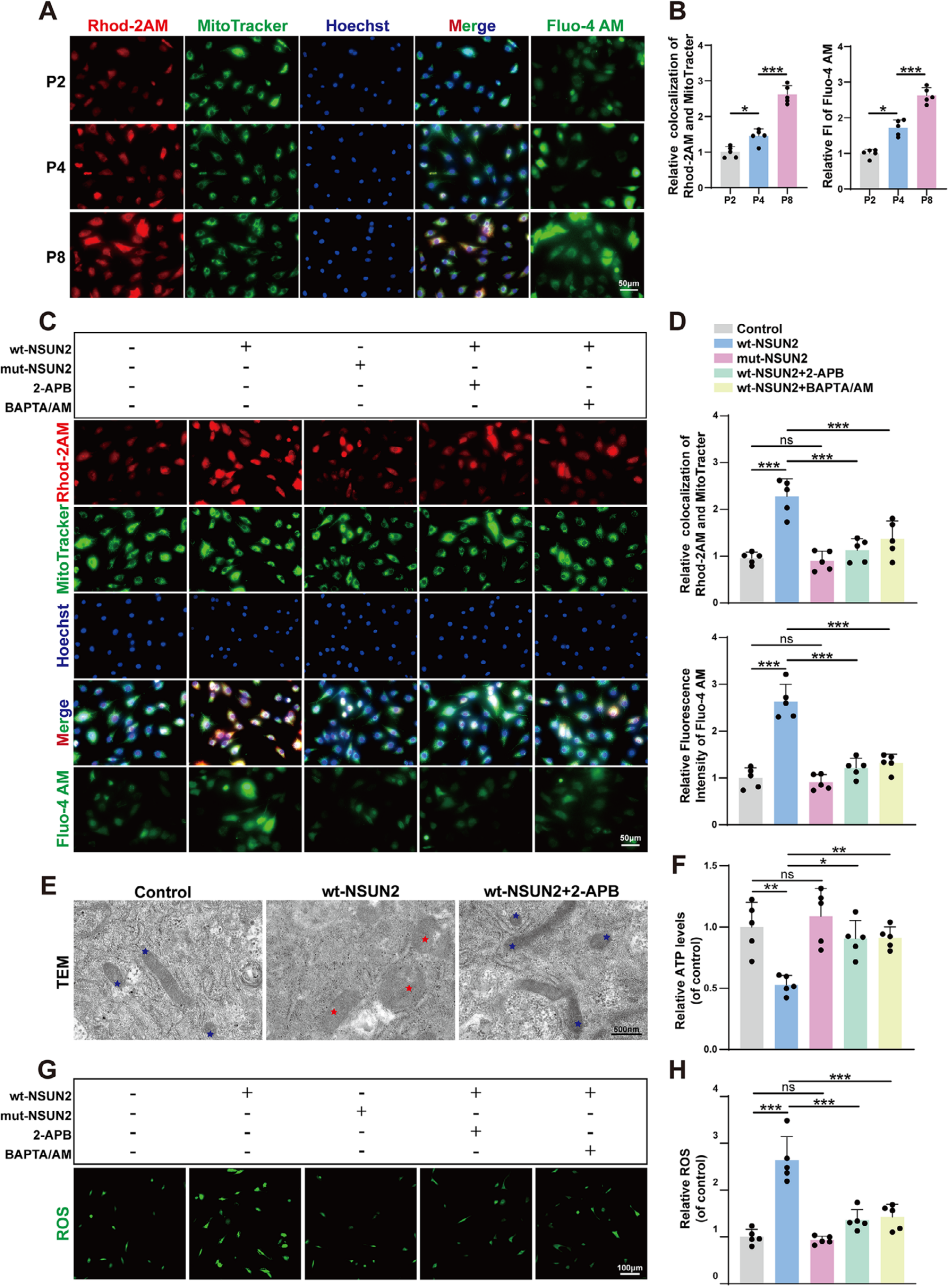
NSUN2 regulates Ca^2+^ flux by IP3R3. (A) Representative IF images of Primary human chondrocytes (HPCs) during passaging after incubation with Rhod‐2 AM and MitoTracker and Fluo‐4 AM. N = 5. bars = 50 µm. (B) Quantifications of fluorescence intensity (FI) of Fluo‐4 AM and colocalization of Rhod‐2 AM and MitoTracker. (C‐H) HPCs were transfected with plasmid encoding wildtype NSUN2 (wt‐NSUN2) or mutant NSUN2 (mut‐NSUN2) then treated by 2‐APB (50 µM) or BAPTA/AM (10 µM) for 24 h. (C) Representative IF images of HPCs incubation with Rhod‐2 AM and MitoTracker and Fluo‐4 AM. N = 5. bars = 50 µm. (D) Quantifications of FI of Fluo‐4 AM and colocalization of Rhod‐2 AM and MitoTracker. (E) Representative TEM images showing mitochondria of HPCs. Blue five‐pointed stars represent normal mitochondria, while red ones represent abnormal mitochondria. N = 3. bars = 500 nm. (F) Relative levels of ATP generation in corresponding groups. (G, H) Intracellular ROS content was detected by DCFH‐DA probe and the Quantifications of fluorescence intensity was analyzed by ImageJ. N = 5. bars = 100 µm. (Data was presented as mean ± SD, ^***^ *p* < 0.001, ^**^ *p* < 0.01, ^*^ *p* < 0.05, ns, no significance).

Given the pivotal role of IP3R3 in forming Ca^2^^+^ channels on mitochondria‐associated membranes and its function in maintaining intracellular Ca^2^^+^ homeostasis [27, 28], we investigated NSUN2's regulatory role in intracellular Ca^2^^+^ homeostasis within chondrocytes. Functional rescue assays were performed. We measured cytosolic Ca^2^^+^ concentration ([Ca^2^^+^]i) and mitochondrial Ca^2^^+^ concentration ([Ca^2^^+^]m) in senescent and OA HPCs using the Fluo‐4 AM and Rhod‐2 AM probes, respectively. The fluorescence intensity (FI) of Fluo‐4 AM and the colocalized FI of Rhod‐2 AM with MitoTracker indicated that [Ca^2^^+^]i and [Ca^2^^+^]m levels were significantly increased in senescent and OA HPCs than in controls (Figure 6A,B, and Figure S5A,B). We performed rescue experiments by treating chondrocytes with 2‐APB and confirmed its efficacy against IP3R3 by WB (Figure S5C,D). Wt‐NSUN2 overexpression increased [Ca^2^^+^]i and [Ca^2^^+^]m levels, which were attenuated by treatment with 2‐APB or BAPTA/AM. mut‐NSUN2 overexpression exhibited no significant effect (Figure 6C,D).

Given the role of [Ca^2^^+^]m overload in impairing mitochondrial function [27, 28], we examined whether NSUN2 modulates mitochondrial function via IP3R3. TEM showed that overexpression of wild‐type NSUN2 induced mitochondrial swelling, vacuolization, and loss of cristae, which were reversed by IP3R3 inhibition with 2‐APB (Figure 6E). Overexpression of wild‐type NSUN2 (but not the mutant form) significantly decreased ATP levels and increased ROS production. Both these effects were rescued by treatment with 2‐APB or BAPTA/AM (Figure 6F–H). These findings suggest that NSUN2 promotes the mitochondrial dysfunction associated with IP3R3‐dependent Ca^2^^+^ dysregulation.

To further provide direct genetic evidence for the role of IP3R3 downstream of NSUN2, we knocked down IP3R3 in NSUN2‐overexpressing chondrocytes (Figure S6A). IP3R3 knockdown markedly attenuated the NSUN2‐induced increase in cytosolic and mitochondrial Ca^2+^ levels, and also reduced SA‐β‐Gal positivity and γH2AX fluorescence intensity (Figure S6B,C). Thus, IP3R3 is a functional downstream effector of NSUN2 that regulates Ca^2+^ homeostasis, inflammasome‐associated activation, and senescence‐related changes in chondrocytes.

### 3.7 NSUN2 Enhances NLRP3 Inflammasome Activation and Senescence‐Associated Phenotypes Through IP3R3‐Mediated Ca^2+^ Flux

**FIGURE 7:**
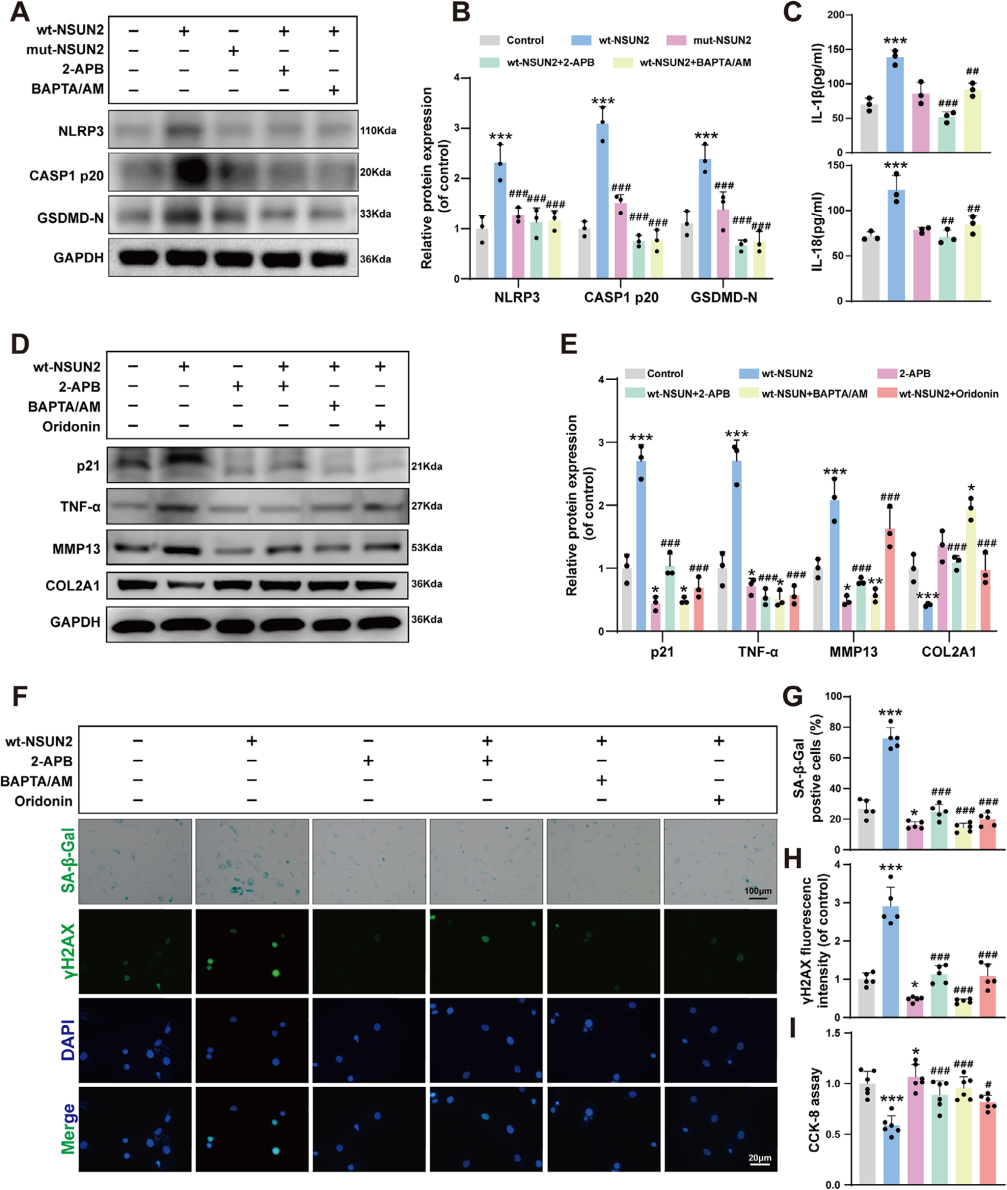
NSUN2 enhances NLRP3 inflammasome activation and senescence‐associated phenotypes through IP3R3‐mediated Ca^2+^ flux. Primary human chondrocytes (HPCs) were transfected with plasmid encoding wildtype NSUN2 (wt‐NSUN2) or mutant NSUN2 (mut‐NSUN2) then treated by 2‐APB (50 µM) or BAPTA/AM (10 µM) for 24 h in A‐C. (A, B) Relative protein expression level and quantification of different Inflammasome‐related genes (NLRP3, CASP1 p20, GSDMD‐N) in HPCs. (C) ELISA analysis of IL‐1β and IL‐18 in supernatants of HPCs. HPCs were transfected with plasmid encoding wildtype NSUN2 (wt‐NSUN2) or control (Vector), then treated with 2‐APB (50 µM) or BAPTA/AM (10 µM) or Oridonin (5 µM) for 24 h in D‐I. (D, E) Relative protein expression level and quantification of p21, TNF‐α, MMP13, COL2A1 in HPCs. (F–H) Representative images and quantification of SA‐β‐Gal and γH2AX staining in HPCs. N = 5. bars = 100 µm (SA‐β‐Gal) or 20 µm (γH2AX). (I) CCK‐8 assay was performed to determine the cell viability in the indicated HPCs. (Data was presented as mean ± SD; ^***^ *p* < 0.001 vs Control, ^###^ *p* < 0.001, ^##^ *p* < 0.01, ^#^ *p* < 0.05 vs wt‐NSUN2‐transfected chondrocytes).

To define the functional consequences of NSUN2–IP3R3‐mediated Ca^2+^ dysregulation, we evaluated phenotypes related to the inflammasome and senescence. In stably transfected chondrocytes, treatment with 2‐APB or BAPTA/AM attenuated the NSUN2‐induced increase in NLRP3, CASP1 p20, and GSDMD‐N levels (Figure 7A,B). The increased secretion of IL‐1β and IL‐18 caused by wt‐NSUN2 overexpression was reduced by 2‐APB or BAPTA/AM (Figure 7C), suggesting that IP3R3‐mediated Ca^2+^ flux contributes to NLRP3 inflammasome activation and downstream pyroptotic signaling‐related changes.

The regulatory mechanisms governing chondrocyte senescence through NSUN2 were also confirmed, besides Ca^2+^ flux inhibition, we treated chondrocytes with oridonin, a NLRP3 inflammasome inhibitor [25, 31], 2‐APB, or BAPTA/AM, which could all counteract the promotional effects on p21, TNF‐α, and MMP13 proteins and the inhibitory effect on COL2A1 protein induced by NSUN2 overexpression (Figure 7D,E). The enhancing effect of SA‐β‐Gal positivity and DNA damage caused by NSUN2 overexpression was compromised by 2‐APB, BAPTA/AM, or oridonin (Figure 7F–H). We explored the effects of NSUN2–IP3R3–inflammasome axis on chondrocyte activity, as shown by the result of CCK8, NSUN2 overexpression decreased chondrocyte activity, which could be counterbalanced by 2‐APB, BAPTA/AM or oridonin (Figure 7I). These results support a model in which NSUN2‐driven IP3R3/Ca^2+^ signaling promotes NLRP3 inflammasome activation, thereby aggravating senescence‐associated phenotypes in chondrocytes.

### 3.8 IP3R3‐Mediated Ca^2+^ Flux Promotes NSUN2 Expression by Targeting SP1

**FIGURE 8:**
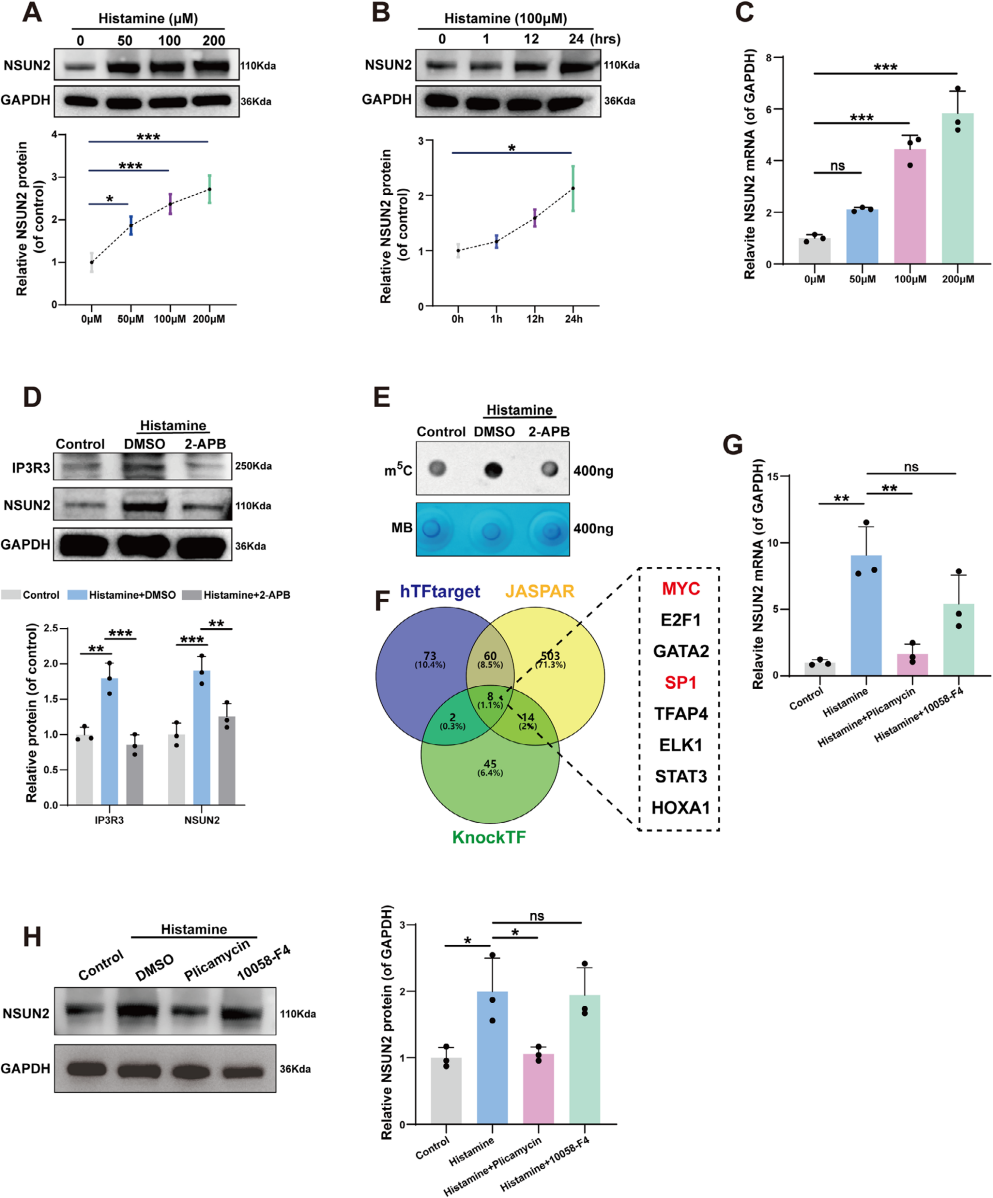
Ca^2+^ promotes the upregulation of NSUN2 expression by targeting SP1, forming a positive feedback loop. (A, B) Relative protein expression level and quantification of NSUN2 in Primary human chondrocytes (HPCs) treated with different concentrations (0, 50, 100, and 200 µM for 24 h) or different durations (100 µM for 0, 1, 12, and 24 h) of histamine. (C) Relative mRNA levels of NSUN2 in HPCs treated with different concentrations (0, 50, 100, and 200 µm for 24 h) of histamine. (D) Relative protein expression level and quantification of IP3R3 and NSUN2 from HPCs treated with histamine following in the presence of DMSO or 2‐APB (50 µM). (E) Relative m^5^C level and quantification in HPCs treated with Histamine following in the presence of DMSO or 2‐APB (50 µM). (F) Eight candidate transcription factors (TFs) were identified through integrated analysis of three prediction databases: JASPAR, hTFtarget, and KnockTF. (G, H) Relative mRNA and protein levels of NSUN2 in HPCs treated with histamine alone or combined with Plicamycin (25 nM) or 10058‐F4 (50 µM) (Data was presented as mean ± SD, ^***^ *p* < 0.001, ^**^ *p* < 0.01, ^*^ *p* < 0.05, ns, no significance).

Because upregulated NSUN2 expression improves Ca^2+^ release from the endoplasmic reticulum (ER), we determined the potential role of Ca^2+^ in NSUN2 upregulation. WB analysis showed that the NSUN2 protein levels in chondrocytes increased with time and concentration following histamine addition, which induces Ca^2+^ release from ER [28] (Figure 8A,B). NSUN2 mRNA levels increased in response to increasing concentrations of histamine (Figure 8C). The increase in NSUN2 protein was attenuated by 2‐APB (Figure 8D). Corresponding changes were also observed in m^5^C levels (Figure 8E). These findings suggested that increased NSUN2 activity stimulates Ca^2+^ release from the ER via IP3R3, thereby establishing a positive feedback mechanism that upregulates NSUN2 expression.

To elucidate the mechanism underlying NSUN2 expression by IP3R3‐mediated Ca^2^^+^ flux, we identified potential transcription factors (TFs) that promote NSUN2 transcription and are regulated by Ca^2+^. Using JASPAR, hTFtarget, and knockTF databases, we intersected the predicted TFs and identified eight candidates (Figure 8F). MYC and SP1 are regulated by Ca^2+^ [32, 33]. Thus, only the SP1 inhibitor, Plicamycin, but not the MYC inhibitor, 10058‐F4, suppressed the histamine‐induced increase in NSUN2 and mRNA levels (Figure 8G,H), suggesting that IP3R3‐mediated Ca^2^^+^ flux upregulates NSUN2 by targeting SP1.

### 3.9 Targeting NSUN2–IP3R3 Axis Alleviates Chondrocyte Senescence and OA Pathogenesis in DMM Mice

**FIGURE 9:**
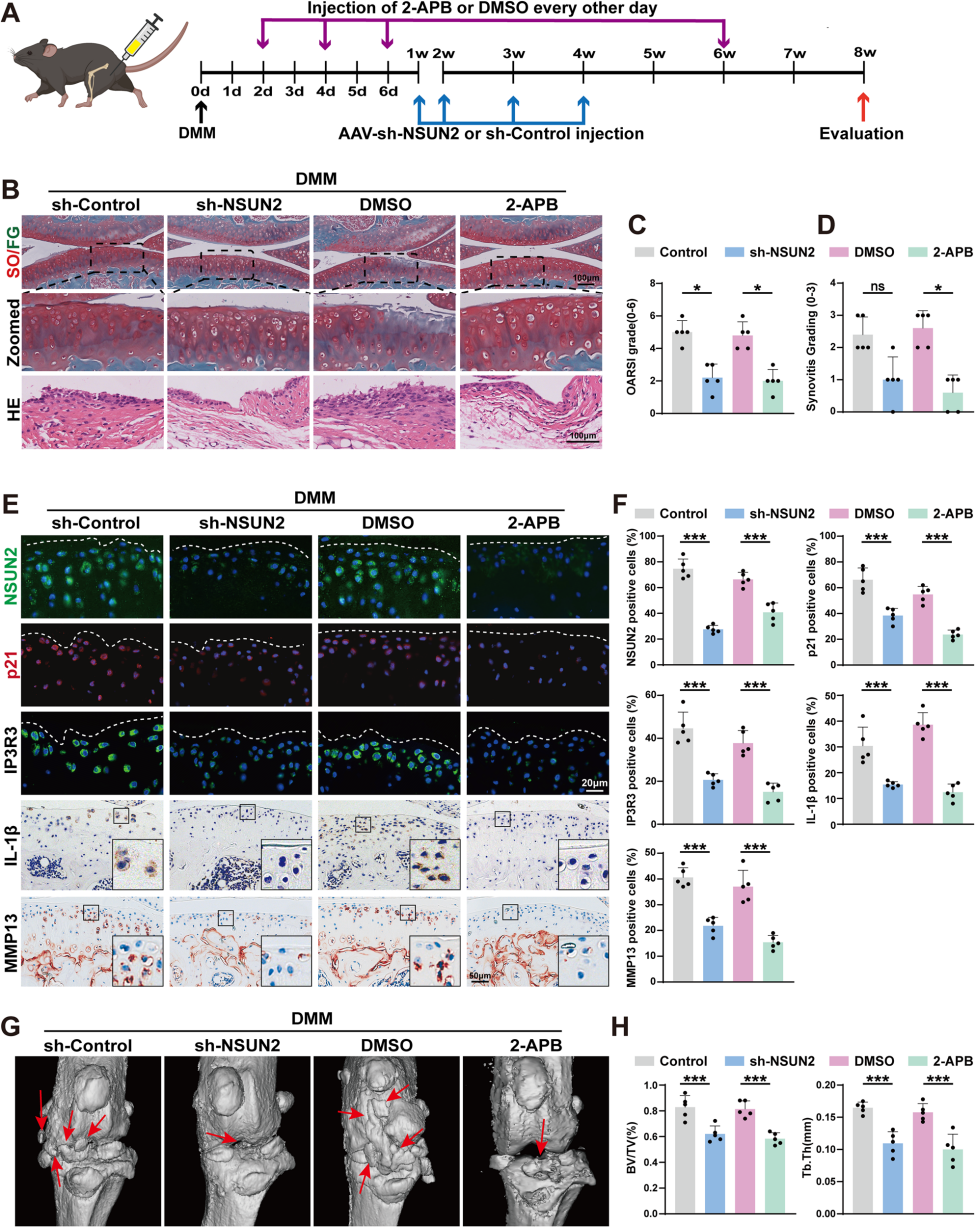
Targeting NSUN2‐IP3R3 signaling alleviates chondrocyte senescence and OA pathogenesis in DMM mice. After 8 weeks of surgical induction, mice in the group of AAV‐sh‐Control (sh‐Control), AAV‐sh‐NSUN2 (sh‐NSUN2), DMSO and 2‐APB were euthanized, and the left hind legs were harvested to perform experiments (N = 5 per group). (A) Schematic illustration of sh‐Control, sh‐NSUN2 and 2‐APB delivery schedules in an DMM‐induced OA model in mice. (B) Representative images of Safranin O/Fast Green staining (SO/FG) in chondrocytes and Hematoxylin‐Eosin (HE) staining in the synovium of the mice knee joints in the corresponding group. bars = 100 µm. N = 5. (C, D) OARSI scores and synovitis grading in four groups of mice. (E) Representative images of immunofluorescence of NSUN2, p21, IP3R3 and immunohistochemistry staining of IL‐1β and MMP13 in chondrocytes of the mice knee joints in the corresponding group. bars = 20 µm or 50 µm. N = 5. (F) Quantitative analysis of NSUN2, p21, IP3R3, IL‐1β, and MMP13 positivity. (G) 3D reconstruction analysis of micro‐CT of the knee joints in mice of the corresponding group. N = 5 per group. (H) BV/TV% was used to detect the relative bone mass of subchondral bone and Tb.Th was used to detect the trabecular thickness of subchondral bone in the mice knee joints. (Data was presented as mean ± SD, ^***^ *p* < 0.001, ^*^ *p* < 0.05, ns, no significance).

To determine the functional role of the NSUN2–IP3R3 axis in OA progression in vivo, we intra‐articularly injected AAV encoding the NSUN2 knockdown plasmid (sh‐NSUN2) or empty plasmid (sh‐Control) and 2‐APB or DMSO into DMM mice (Figure 9A). The sh‐NSUN2 group exhibited reduced cartilage destruction, as evidenced by smoother articular surfaces, increased cartilage thickness, fewer cartilage fissures and erosions, enhanced matrix and chondrocyte presence, lower OARSI scores, and diminished synovitis grading than in the sh‐control group. 2‐APB treatment produced chondroprotective effects similar to those of NSUN2 knockdown, as demonstrated by comparison with DMSO‐treated mice (Figure 9B–D). IHC and IF analyses showed that positivity staining of NSUN2, IP3R3, IL‐1β, MMP13, and p21 decreased, and positivity staining of COL2A1 increased in sh‐NSUN2 and 2‐APB‐treated mice than in controls (Figure 9E,F). Micro‐CT 3D reconstruction showed that cartilage osteophyte formation in the knee joints was significantly decreased in sh‐NSUN2‐ and 2‐APB‐treated mice than in control mice (Figure 9G). Subchondral bone sclerosis decreased, and the bone trabeculae were thinner in sh‐NSUN2‐ and 2‐APB‐treated mice than in controls, as assessed based on BV/TV and Tb.Th (Figure 9H) [34]. These findings suggest that the NSUN2–IP3R3 pathway may be a promising target for OA treatment, potentially slowing chondrocyte aging and inhibiting inflammation.

## 4 Discussion

**FIGURE 10:**
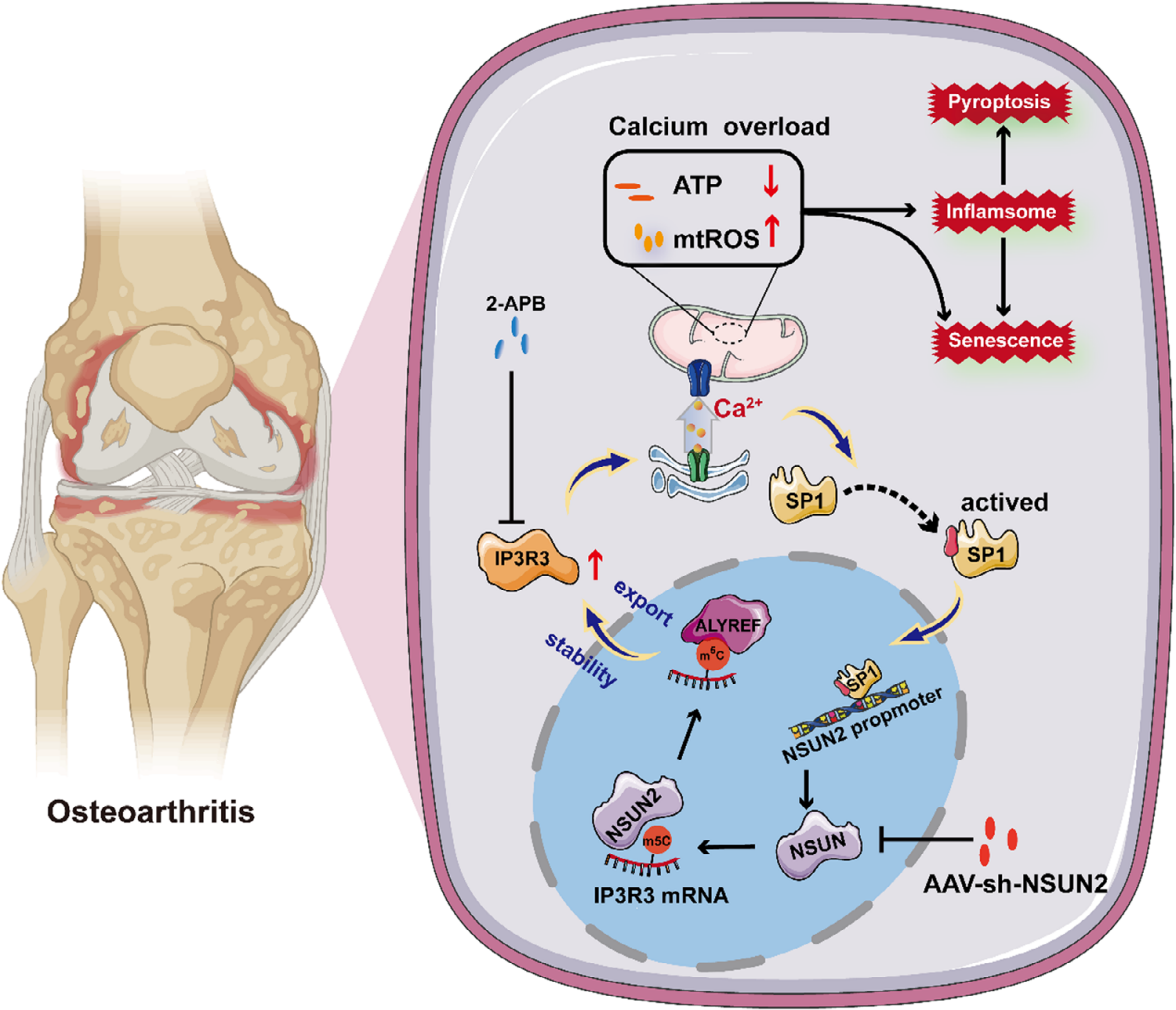
A proposed model illustrating the Ca^2+^/NSUN2/IP3R3 regulatory pathway‐mediated NLRP3 inflammasome in promoting senescence of chondrocytes during OA progression.

In the present study, we identified NSUN2 and m^5^C modifications as crucial regulators of OA development. Mechanistically, NSUN2, in combination with ALYREF, increases m^5^C modification, promotes export, and stabilizes IP3R3 mRNA, thus activating NLRP3 inflammasome signaling by promoting Ca^2+^ overload (Figure 10). The mutation of the m^5^C deposition sites on NSUN2 or the m^5^C recognition sites on ALYREF inhibited the regulatory effect on IP3R3. NSUN2 knockdown or pharmacological inhibition of Ca^2+^ channels protect chondrocytes against senescence and OA progression in vitro and in vivo.

Senescence is an important driver of OA progression. m^5^C modification exerts biological effects, including roles in inflammation, aging, and stress responses [35], whereas NSUN2 mediates the oxidative stress‐induced expression of p16INK4a, p21, and p53 [14]. Although direct evidence associating NSUN2 with chondrocyte senescence and inflammation is limited, previous studies reported significant differences in the number and distribution of m^5^C peaks between OA and normal cartilage tissues [18], indicating that m^5^C modifications contribute to cartilage aging and OA progression. We observed increased m^5^C modification and NSUN2 expression in senescent chondrocytes and in cartilage tissues from patients with OA and DMM mice. Gain‐ and loss‐of‐function experiments showed that NSUN2 promotes chondrocyte senescence and OA progression in vitro and in vivo. Notably, mutations in the catalytic cysteine residues, C271 and C321, considerably attenuated these effects, suggesting that the prosenescent role of NSUN2 depends on its m^5^C methyltransferase activity.

To determine how increased NSUN2‐mediated m^5^C modification promotes chondrocyte senescence, we performed RNA sequencing and identified the NLRP3 inflammasome pathway as an important downstream signaling axis. NLRP3 is implicated in age‐related inflammation and OA progression, and its pharmacological inhibition exhibits protective effects across multiple age‐associated disease contexts [36, 37, 38, 39, 40]. Consistent with these observations, we found that NLRP3 inflammasome‐related markers increased in senescent chondrocytes and OA cartilage, and oridonin was attenuated senescence‐associated changes. These findings suggest a functional association between NLRP3 inflammasome activation and chondrocyte senescence during OA progression.

We identified IP3R3 mRNA as a potential downstream target of NSUN2 by m^5^C MeRIP‐sequencing and subsequent MeRIP‐qRT‐PCR and RIP‐qRT‐PCR analyses. IP3R3 is an ER‐associated Ca^2+^ release channel that plays an important role in intracellular Ca^2+^ homeostasis, and excessive IP3R3 activity is associated with Ca^2+^ overload, mitochondrial dysfunction [27, 28], NLRP3 inflammasome activation [21, 41], aging [42], even cell death [28, 43]. Ca^2+^ dysregulation contributes to OA progression [44, 45, 46]. The cytosolic Ca^2+^ concentration ([Ca^2+^]i) and mitochondrial Ca^2+^ concentration ([Ca^2+^]m) were increased in OA and senescent chondrocytes. Functionally, NSUN2 overexpression is accompanied by increased Ca^2+^ accumulation and downstream pathological changes, whereas these phenotypes are attenuated by pharmacological modulation of Ca^2+^ signaling. Together with NSUN2‐dependent regulation of IP3R3 expression, these findings support the role of the IP3R3/Ca^2+^ axis in linking NSUN2 to inflammasome‐associated activation and senescence‐related phenotypes in chondrocytes. This is partially supported by pharmacological rescue experiments using 2‐APB and BAPTA/AM. Although these agents consistently attenuated NSUN2‐induced Ca^2+^ dysregulation and downstream phenotypic changes, they were not fully pathway‐specific. Specifically, 2‐APB may affect Ca^2+^ signaling through targets other than IP3R3, whereas BAPTA/AM functions as a broader intracellular Ca^2+^ chelator. Hence, these findings should be interpreted as supportive evidence for the involvement of the IP3R3/Ca^2+^‐associated pathway, rather than as definitive proof of complete target specificity at the IP3R3 level. This interpretation is consistent with recent OA studies showing that chondroprotective interventions can converge on inflammation‐, senescence‐, and stress‐related pathways, including vaccarin‐mediated suppression of the JNK/SAA2 axis and CORM‐3‐mediated regulation of MAPK/mTOR‐associated inflammatory and autophagic responses [47, 48].

Although cellular senescence, inflammasome activation, and pyroptotic signaling are mechanistically linked during OA progression, they should not be considered interchangeable. We primarily characterized senescence by SA‐β‐Gal positivity, γH2AX, p16/p21, and matrix/SASP‐associated changes, whereas inflammasome activation was defined by NLRP3 pathway‐associated events, including NLRP3, NEK7 colocalization, CASP1 activation, and IL‐1β/IL‐18 maturation. Increases in GSDMD‐N, together with the Ac‐YVAD‐cmk‐sensitive rescue of NSUN2‐induced viability loss, were interpreted as evidence of pyroptotic signaling‐related changes downstream of inflammasome activation. This distinction is important for interpreting our findings, as NSUN2 links senescence‐associated chondrocyte dysfunction with NLRP3‐driven inflammatory responses via the IP3R3/Ca^2+^ axis. Furthermore, NSUN2‐driven IP3R3/Ca^2+^ dysregulation preferentially affected the NLRP3 inflammasome but not the AIM2 or NLRC4 inflammasomes in our chondrocyte model. Although Ca^2+^ overload and mitochondrial ROS represent types of intracellular stress, NLRP3 is well recognized as a stress‐responsive inflammasome that integrates ionic perturbations, mitochondrial dysfunction, and ROS‐related danger signals. However, AIM2 activation requires cytosolic double‐stranded DNA, whereas NLRC4 activation depends on NAIP‐mediated recognition of bacterial ligands, including flagellin or type III secretion system components [49, 50]. Therefore, during sterile OA‐associated chondrocyte stress, the NSUN2–IP3R3 axis could create a cellular state permissive for NLRP3 activation while remaining insufficient to engage AIM2 or NLRC4 in the absence of their more specific upstream triggers.

This study has some limitations. Although the pharmacological rescue experiments using 2‐APB, BAPTA/AM, and oridonin provide consistent support for the role of the IP3R3/Ca^2^^+^/NLRP3‐associated pathway, these compounds are pharmacological tools with known limitations, including incomplete specificity and potential off‐target effects. Because intra‐articular AAV5 delivery used in the in vivo rescue experiment was not cell type‐specific, NSUN2 knockout mice would be valuable for further confirming the role of NSUN2 in cartilage aging and OA progression in vivo. We did not screen for direct small‐molecule inhibitors of NSUN2, which is an important step in future translational development. Although our results suggest that Ca^2^^+^ promotes NSUN2 expression, at least in part, through SP1, the mechanistic depth of this conclusion remains limited. SP1 is considered a candidate mediator. The upstream signaling events and transcriptional mechanisms associating Ca^2^^+^ flux with NSUN2 upregulation require further investigation.

To conclude, these findings elaborate on the role of the NSUN2‐m^5^C–IP3R3 axis in the chondrocyte NLRP3 inflammasome and senescence and how its disruption contributes to OA progression. NSUN2 and its modification of the m^5^C‐ and IP3R3‐mediated Ca^2+^ flux can be novel targets for OA treatment.

## Author Contributions

**Guping Mao**: funding acquisition, validation, project administration, writing – review & editing. **Wei Li**: investigation, methodology, data curation, formal analysis, writing – original draft. **Zhencan Lin**: methodology, formal analysis. **Zengfa Deng**: methodology, formal analysis. **Ming Li**: funding acquisition, software. **Zongrui Jiang**: methodology. **Changzhao Li**: funding acquisition, writing – review & editing. **Dianbo Long**: methodology, investigation. **Yan Kang**: supervision, conceptualization, writing – review & editing.

## Funding

This study was supported by National Natural Science Foundation of China (82572757), Guangdong Basic and Applied Basic Research Foundation Natural Science Fund Project‐ Youth Promotion Project (2024A1515030260), Natural Science Foundation of Guangdong Province (2025A1515012420; 2026A1515012820; 2026A1515012724), Fundamental Research Funds for the Central Universities, Sun Yat‐sen University (80000‐31610012), National Natural Science Foundation of China (82202739), National Natural Science Foundation of China (81972051).

## Conflicts of Interest

The authors declare no conflicts of interest.

## Supporting information




**Supporting File**: advs76370‐sup‐0001‐SuppMat.docx.

## Data Availability

The data that support the findings of this study are available from the corresponding author upon reasonable request.
