# Supplementary material for: Targeting NSUN2‐Mediated m5C Modification Attenuates Chondrocyte Senescence and NLRP3 Activation in Osteoarthritis
Source: Adv Sci (Weinh). 2026 Jul 6:e76370. Online ahead of print. doi: 10.1002/advs.76370 (PMC13335690; doi:10.1002/advs.76370)
Supplement: Supplementary file 1 — Supporting File: advs76370‐sup‐0001‐SuppMat.docx. [file ADVS-9999-e76370-s001.docx]

**Supplementary Figures**


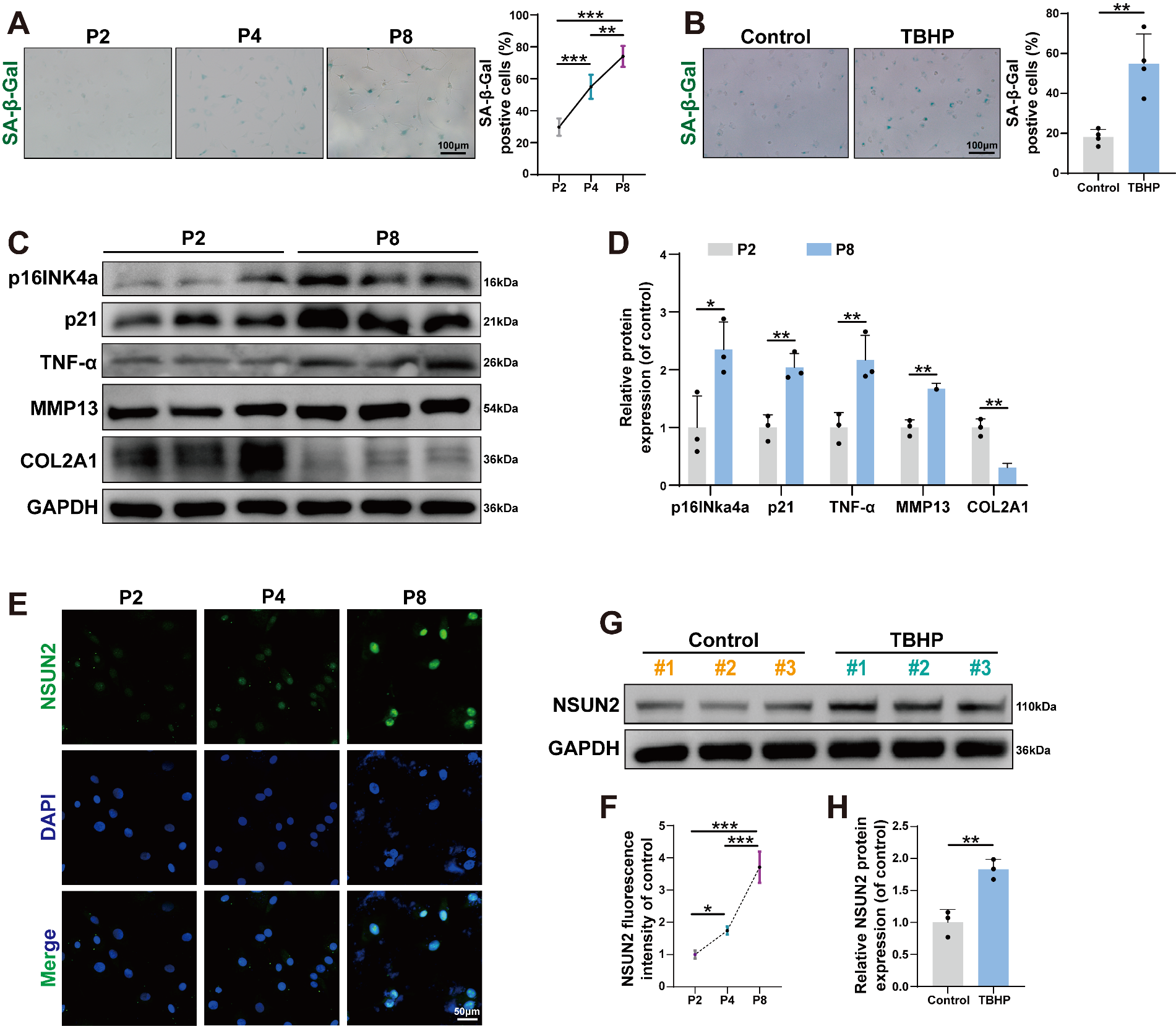


**Fig. S1. m^5^C level and methyltransferase NSUN2 expression are increased in senescent chondrocytes and OA cartilage tissue.** (**A, B**) Images and quantification of SA-β-Gal positivity in human primary chondrocytes (HPCs) at passage 2,4 and 8 (N = 3), and treated by TBHP (100 μM) or not (N = 5), bars = 100 μm. (**C, D**) The protein expression levels and quantification of p16INK4a, p21, TNF-α, MMP13, COL2A1 in HPCs at passage 2 and 8. **(E, F**) Representative images and quantification of immunofluorescence staining of NSUN2 during passaging of HPCs. N = 3, bars = 50 μm. (**G, H**) The expression of NSUN2 in HPCs treated or untreated by TBHP (100 μM) for 24 h. N = 3. (Data was manifested as mean ± SD, ^***^p < 0.001, ^**^p < 0.01, ^*^p < 0.05).


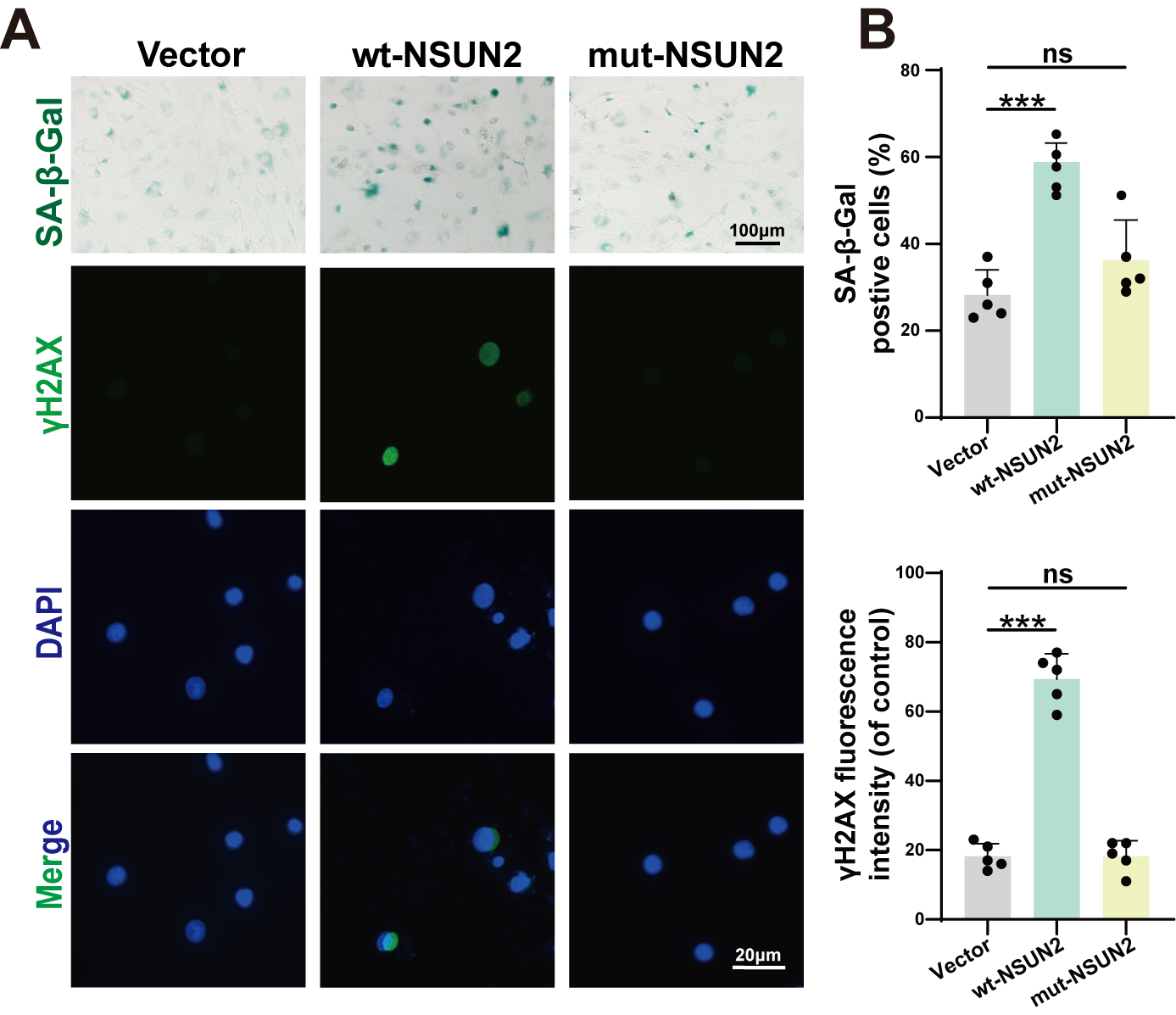


**Fig. S2. NSUN2 promote cellular senescence by m^5^C in chondrocytes.** (**A, B**) Images and quantification of SA-β-Gal and IF staining of γH2AX in human chondrocytes at passage 2 transfected with Vector, wt-NSUN2, mut-NSUN2. N = 5, bars = 100 (SA-β-Gal) or 20 μm (γH2AX). (Data was manifested as mean ± SD, ^***^p < 0.001, ns, no significance).


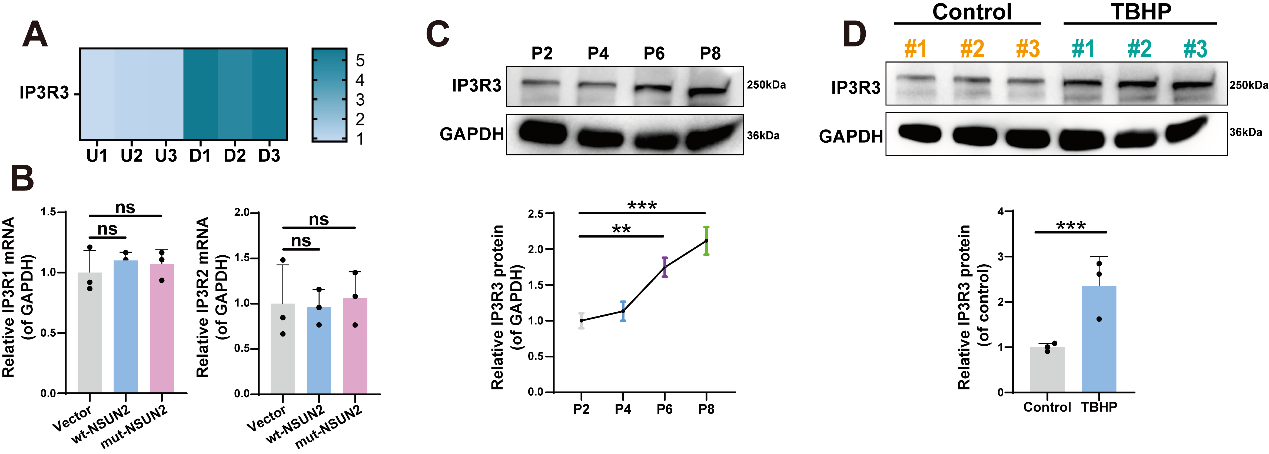


**Fig. S3. IP3R3 is highly expressed in aged chondrocytes.** (**A**) RNA-seq showing the expression of IP3R3 in intact and damaged articular cartilage sections collected from OA patients. (**B**) Relative mRNA of IP3R1 and IP3R2 when wt-NSUN2 or mut-NSUN2 was overexpressed in human primary chondrocytes (HPCs). (**C, D**) Relative protein levels of IP3R3 in HPCs during passaging and treated by TBHP (100 μM) for 24 h or not. N = 3 (Data was manifested as mean ± SD, ^***^p < 0.001, ^**^p < 0.01, ns, no significance).


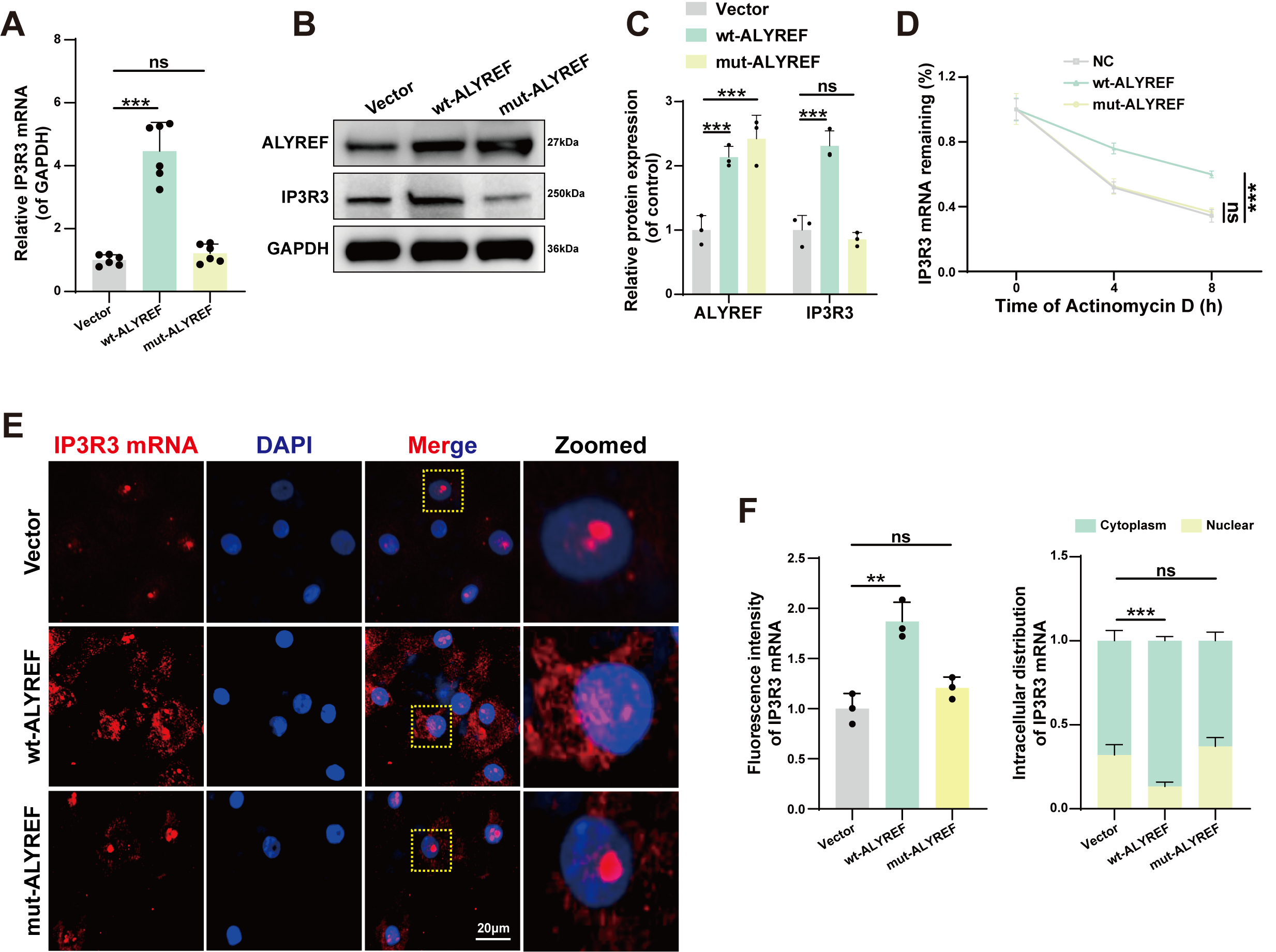


**Fig. S4. ALYREF facilitates export by m^5^C modification.** (**A-E**) Primary human chondrocytes (HPCs) were transfected with plasmid encoding wildtype ALYREF (wt-ALYREF) or mutant ALYREF (mut-ALYREF) or plasmid control with no insert (Vector). (**A**) The relative IP3R3 mRNA levels in HPCs. **(B, C)** Relative protein expression level of IP3R3 in HPCs. (**D**) RNA stability assay showing IP3R3 mRNA half-life in HPCs, N = 3. (**E, F**) Representative fluorescence in situ hybridization images and quantifications showed that the distribution of IP3R3 mRNA in chondrocytes. N = 3, bars = 20 μm. (Data was manifested as mean ± SD, ^***^p < 0.001, ^**^p < 0.01, ns, no significance).


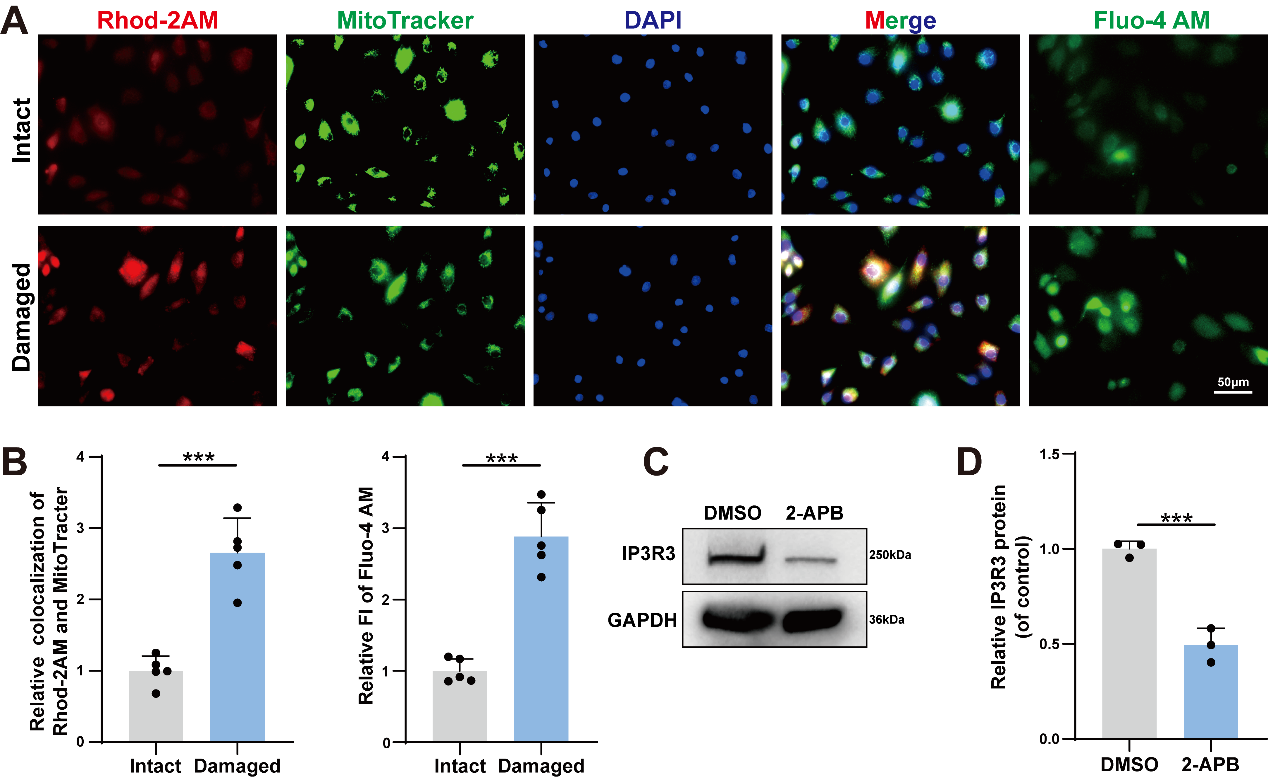


**Fig. S5. Chondrocytes of damaged cartilage have higher Ca^2+^ flux than intact cartilage cells.**

**(A, B)** Representative fluorescence intensity (FI) images and quantifications of chondrocytes of intact and damaged articular cartilage incubation with with FLuo-4 AM, or Rhod-2 AM and MitoTracker. N = 5. bars = 50 μm. **(C, D)** Relative IP3R3 protein expression level and quantification in chondrocytes treated by 2-APB (50 μM) or DMSO for 24 h. (Data was manifested as mean ± SD, ^***^p < 0.001).


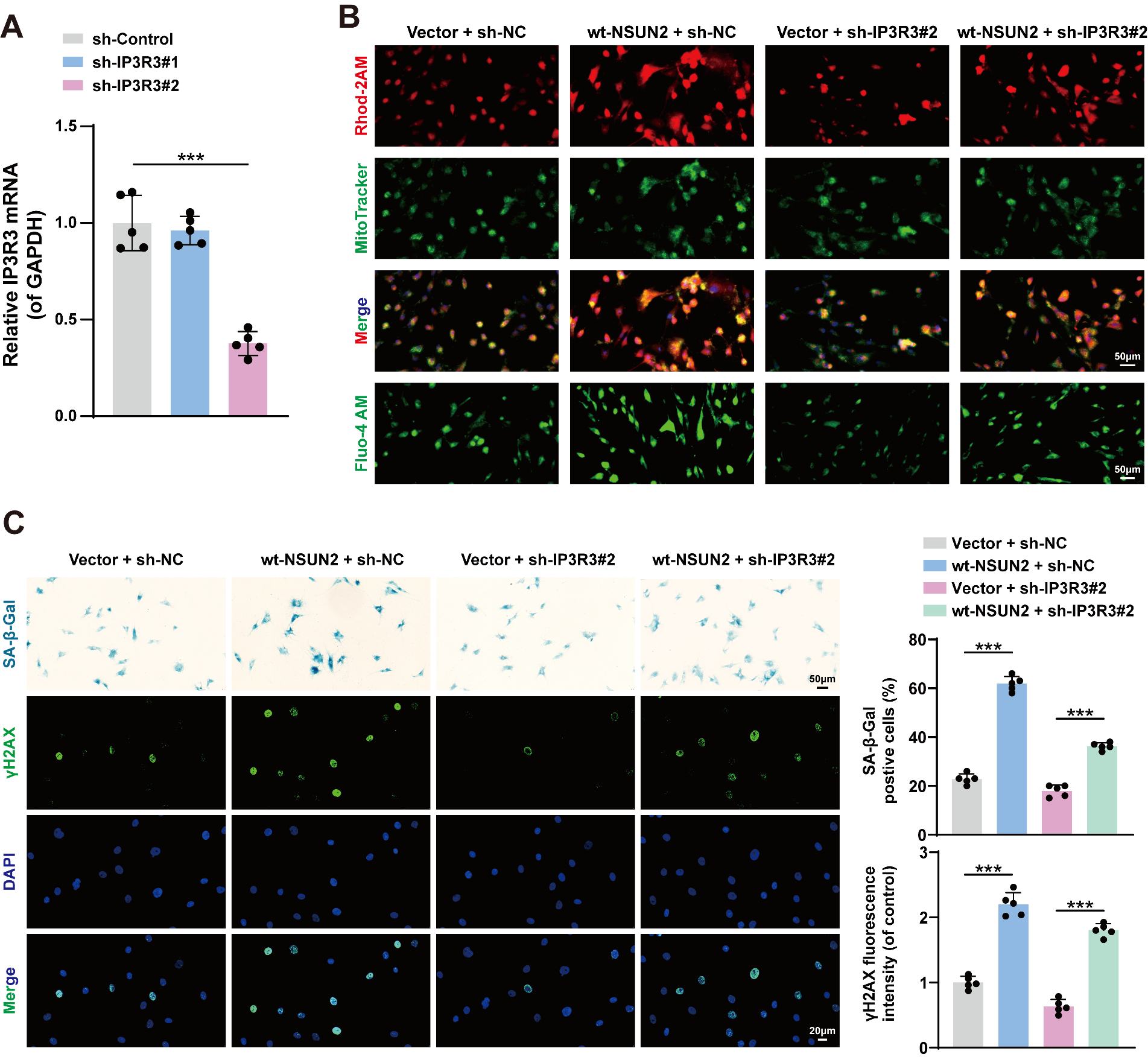


**Fig. S6. Genetic knockdown of IP3R3 attenuates NSUN2-induced Ca²⁺ accumulation and senescence-associated phenotypes in chondrocytes. (A)** qRT-PCR analysis of IP3R3 knockdown efficiency in chondrocytes transfected with sh-NC, sh-IP3R3#1, or sh-IP3R3#2. sh-IP3R3#2 was selected for subsequent rescue experiments. N = 5. **(B)** Representative fluorescence images showing cytosolic Ca²⁺ levels detected by Fluo-4 AM and mitochondrial Ca²⁺ accumulation assessed by Rhod-2 AM/MitoTracker colocalization in chondrocytes transfected with Vector or wt-NSUN2 together with sh-NC or sh-IP3R3#2. Scale bar = 50 μm. **(C)** Representative images and quantification of SA-β-Gal staining and γH2AX immunofluorescence in chondrocytes transfected with Vector or wt-NSUN2 together with sh-NC or sh-IP3R3#2. N = 5. Scale bars = 50 μm for SA-β-Gal and 20 μm for γH2AX. (Data was manifested as mean ± SD, ^***^p < 0.001).

**Table S1: Primer sequences for qRT-PCR**

| **Gene** | **Primer sequences** |
| --- | --- |
| NSUN2  (human) | Forward: CAAGCTGTTCGAGCACTACTAC  Reverse: CTCCCTGAGAGCGTCCATGA |
| GAPDH  (human) | Forward: AGGTCGGTGTGAACGGATTTG  Reverse: GGGGTCGTTGATGGCAACA |
| IP3R3  (human) | Forward: CCAAGCAGACTAAGCAGGACA  Reverse: ACACTGCCATACTTCACGACA |
| IP3R1  (human) | Forward: ATTGCTGGGGACCGTAATCC  Reverse: TCCAATGTGACTCTCATGGCA |
| IP3R2  (human) | Forward: CACCTTGGGGTTAGTGGATGA  Reverse: CTCGGTGTGGTTCCCTTGT |

**Table S2: The sequences of shRNAs**

| **shRNA** | **Sequence (5′-3′)** |
| --- | --- |
| sh-NSUN2#1 | GAGCGATGCCTTAGGATATTA |
| sh-NSUN2#2 | CAGTGGAAGGTAATGACGAAA |
| Sh-ALYREF | GAACTCTTTGCTGAATTTGGA |

| **Table S3. Baseline characteristics of patients with osteoarthritis (n = 32).** | |
| --- | --- |
| **Variables** | **Counts (%)** |
| **Age** |  |
| <65 | 12 (37.5) |
| ≥65 | 20 (62.5) |
| **Sex** |  |
| Male | 6 (18.7) |
| Female | 26 (81.3) |
| **Body Mass Index** |  |
| <18.5 | 0 (0.0) |
| ≥18.5, <24 | 8 (25.0) |
| ≥24, <28 | 16 (50.0) |
| ≥28 | 8 (25.0) |
| **Affected side** |  |
| Left | 14 (43.8) |
| Right | 18 (56.3) |
| **Disease duration (years)** |  |
| <10 | 15 (46.9) |
| ≥10 | 17 (53.1) |
| **Kellgren–Lawrence grade** |  |
| III | 12 (37.5) |
| IV | 20 (62.5) |
